# Structural Modifications Yield Novel Insights Into the Intriguing Pharmacodynamic Potential of Anti-inflammatory Nitro-Fatty Acids

**DOI:** 10.3389/fphar.2021.715076

**Published:** 2021-11-18

**Authors:** Nadine Hellmuth, Camilla Brat, Omar Awad, Sven George, Astrid Kahnt, Tom Bauer, Hai Phong Huynh Phuoc, Dieter Steinhilber, Carlo Angioni, Mohamed Hassan, Katharina J. Hock, Georg Manolikakes, Kai Zacharowski, Jessica Roos, Thorsten J. Maier

**Affiliations:** ^1^ Department of Anesthesiology, Intensive Care Medicine and Pain Therapy, University Hospital Frankfurt, Goethe-University, Frankfurt, Germany; ^2^ Paul-Ehrlich Institute, Federal Institute for Vaccines and Biomedicines, Langen, Germany; ^3^ Institute of Pharmaceutical Chemistry, Goethe-University, Frankfurt, Germany; ^4^ Pharmazentrum Frankfurt/ZAFES, Institute of Clinical Pharmacology, Goethe-University, Frankfurt, Germany; ^5^ Department of Chemistry, TU Kaiserslautern, Kaiserslautern, Germany; ^6^ Department of Chemistry, Faculty of Science, Aswan University, Aswan, Egypt

**Keywords:** nitroalkene, michael acceptor, structure-function, NF-κB, soluble epoxide hydrolase, Nrf-2, 5-lipoxygenase, cyclooxygenase-2

## Abstract

Endogenous nitro-fatty acids (NFA) are potent electrophilic lipid mediators that exert biological effects *in vitro* and *in vivo via* selective covalent modification of thiol-containing target proteins. The cytoprotective, anti-inflammatory, and anti-tumorigenic effects of NFA in animal models of disease caused by targeted protein nitroalkylation are a valuable basis for the development of future anti-phlogistic and anti-neoplastic drugs. Considering the complexity of diseases and accompanying comorbidities there is an urgent need for clinically effective multifunctional drugs. NFA are composed of a fatty acid backbone containing a nitroalkene moiety triggering Michael addition reactions. However, less is known about the target-specific structure–activity relationships and selectivities comparing different NFA targets. Therefore, we analyzed 15 NFA derivatives and compared them with the lead structure 9-nitro-oleic acid (9NOA) in terms of their effect on NF-κB (nuclear factor kappa B) signaling inhibition, induction of Nrf-2 (nuclear factor erythroid 2-related factor 2) gene expression, sEH (soluble epoxide hydrolase), LO (lipoxygenase), and COX-2 (cyclooxygenase-2) inhibition, and their cytotoxic effects on colorectal cancer cells. Minor modifications of the Michael acceptor position and variation of the chain length led to drugs showing increased target preference or enhanced multi-targeting, partly with higher potency than 9NOA. This study is a significant step forward to better understanding the biology of NFA and their enormous potential as scaffolds for designing future anti-inflammatory drugs.

## 1 Introduction

In the last decade, nitro-fatty acids (NFA) have attracted increasing attention because they represent anti-inflammatory, cytoprotective, and anti-tumorigenic endogenous mediators that showed therapeutic effects in different animal models of disease and safety in phase I clinical trials (Piesche et al., 2020). NFA have been found in the plasma of healthy humans at nano to picomolar concentration levels (Baker et al., 2005; Tsikas et al., 2009). Furthermore, they are ingredients in the Mediterranean diet (Fazzari et al., 2014), and the dietary supplementation of unsaturated fatty acids in combination with nitrite or nitrate can alter plasma NFA levels (Delmastro-Greenwood et al., 2015). Additionally, NFA can be generated endogenously in tissues under inflammatory conditions by reactions of reactive nitrogen species with unsaturated fatty acids (Freeman et al., 2008). Electrophilic NFA contain a Michael acceptor moiety targeting nucleophilic amino acids, preferably thiols of cysteines, but also histidine, imidazole and lysine ε-amino groups. Upon Michael addition, these nucleophilic targets become reversibly nitroalkylated, leading to post-translational protein modifications, regulating numerous signaling pathways (Batthyany et al., 2006; Baker et al., 2007).

NFA modulate nuclear factor kappa B (NF-κB) signaling by nitroalkylation of both transcription factor subunits p65 and p50 leading to the inhibition of transcription factor translocation and the reduction of DNA-binding affinity (Villacorta et al., 2013; Khoo et al., 2018). NFA also trigger translocation of the cytoprotective transcription factor nuclear factor erythroid 2-related factor 2 (Nrf-2) into the nucleus and activation of antioxidant gene expression, including GSH (glutathione) and HO-1 (heme oxygenase 1). Translocation of Nrf-2 is induced by nitroalkylation of Kelch-like ECH-associated protein 1 (Keap1) and subsequent dissociation from Nrf-2. (Kobayashi and Yamamoto, 2005; Cole et al., 2009; Kansanen et al., 2009; Tsujita et al., 2011). NFA also target soluble epoxide hydrolase (sEH) playing a key role in regulating blood pressure, as it catalyzes the hydration of epoxyeicosatrienoic acid (EET) to the less active dihydroxyeicosatrienoic acid (DHET), thereby modulating EET-dependent blood vessel ton. NFA target cysteine 521 in the catalytic center of sEH, leading to loss of function *in vitro* and *in vivo*, as shown in a hypertension mouse model (Charles et al., 2011; Charles et al., 2014). Furthermore, NFA inhibit 5-lipoxygenase (5-LO), the key enzyme in leukotriene biosynthesis, via the nitroalkylation of cysteine 416 and 418. Direct inhibition of 5-LO by NFA efficiently reduced pulmonary inflammation in mice (Awwad et al., 2014; Maucher et al., 2017). In addition, nitro-AA (nitro-arachidonic acid) inhibits cyclooxygenase COX-1 and COX-2 peroxidase activity as well as COX-1 oxygenase thereby causing suppression of prostaglandin formation and inhibition of platelet activation *in vivo* in a PKCα (protein kinase C alpha)-dependent manner (Malkowski, 2000; Trostchansky et al., 2011; Bonilla et al., 2013; Rubbo, 2013; Wood et al., 2019). NFA also serve as peroxisome proliferator-activated receptor (PPAR) agonists (Baker et al., 2005; Schopfer et al., 2005) exerting neuroprotective effects in multiple animal models such as Parkinson’s and Alzheimer’s disease as well as multiple sclerosis (SUNDARARAJAN et al., 2006).

The different targets of NFA can explain their broad spectrum of therapeutic effects. Not only do NFA initiate processes of inflammatory resolution, but they also confer protective effects against cardiovascular diseases, as they exert anti-hypertensive effects (Charles et al., 2011), as well as reducing the infarct size in models of atherosclerosis (Villacorta, 2016), and induce vasorelaxation (Freeman et al., 2008). Protective effects of NFA against fibrosis have also been recently discovered (Rom et al., 2019). The important role of NFA in tumorigenesis is currently under investigation. NFA inhibition of breast cancer growth based on NF-κB signaling modulation has been observed (Woodcock et al., 2018). Previously, we demonstrated in a colorectal cancer cell xenograft animal model the suppression of tumor growth caused by mitochondrial dysfunction–mediated apoptosis (Kühn et al., 2018).

NFA metabolism leads to the formation of diverse secondary species with potentially different tissue and organ distribution and altered pharmacokinetic and pharmacodynamic profiles. NFA are substrates for ß- and ω-oxidation and are esterified into complex lipids (Rudolph et al., 2009; Fazzari et al., 2015; Salvatore et al., 2017). Most of the resulting metabolites retain their electrophilic reactivity unless they become reduced to non-electrophilic fatty acids by prostaglandin reductase-1 (PtGR-1) (Vitturi et al., 2013). ß-oxidation of NFA reduces fatty acid chain length up to eight carbon atoms, leading to a progressive rise in their polarity. Therefore, shortening NFA may promote their reactivity in polar environments. However, esterification enables the incorporation of NFA into biological membranes. This can prevent electrophilic lipids from reacting with intracellular thiols, contemporaneously generating a NFA depot (Schopfer et al., 2018). Thus, studies investigating NFA metabolites have provided first evidence that structural modification may have a relevant impact on the biological effects of these mediators. However, less is known about the pharmacological effects of NFA metabolites.

From a pharmacological point of view selectivity and potency of the Michael acceptor function of NFA might be changed with different drug design strategies such as: substitution of the electron-withdrawing group to adjust electrophilicity, alteration of the chain length and Michael acceptor position to potentially improve accessibility to the target molecule, and the addition of different terminal groups. However, less is known about the pharmacological effects of such NFA derivatives with systematically modified structures. First evidence was provided by Khoo *et al.*, who investigated the biological effects of NFA derivatives using luciferase-based reporter gene assays (Khoo et al., 2018).

In the present study, we hypothesized that changing the Michael acceptor position and chain length may alter the affinity of NFA to their respective targets, potentially allowing more selective effects and rendering these derivatives novel drug candidates.

We, therefore, used sophisticated methods to investigate the effects of a series of NFA derivatives regarding their modulatory potency and efficacy on the NF-κB, Nrf-2, 5-LO, sEH, and COX-2 pathways and their cytotoxicity in colorectal cancer cells. Here, we show that alteration of the chain length and Michael acceptor position has a large impact on NFA binding to the respective target proteins, allowing more potent and selective effects of NFA derivatives on selected signaling pathways.

## 2 Materials and Methods

### 2.1 Cell Culture and Reagents

If not indicated otherwise, all reagents were purchased from Cayman Chemicals (Ann Arbor, Michigan, United States) at reagent grade. 9-NOA and all other NFA derivatives in this study were synthesized according to previously established methods (Hock et al., 2017; Hassan et al., 2021) and analyzed by ^1^H-NMR, ^13^C-NMR and ESI-MS. Starting from 9-NOA as chemical lead 15 NFAs with different positioning of the nitroolefin and variation of the overall chain length were prepared. Their structures are illustrated in Tables 1, 2. The spectral data of all NFA derivatives prepared for this study match those of the previously reported compounds. All NFA derivatives were purified by preparative MPLC prior to biological studies. All compounds showed a purity >95% as determined by ^1^H-NMR and ^13^C-NMR. The E/Z-ratio of all NFA derivatives was determined by ^1^H-NMR (based on the integrals of the olefinic proton signals). Eukaryotic cell lines were obtained from DSMZ (German Collection of Microorganisms and Cell Cultures, Braunschweig, Germany) unless otherwise specified. HCT-116 (DSMZ no.: ACC 58, human colon carcinoma), HT-29 (DSMZ no.: ACC 299, human colon adenocarcinoma), A549 (DSMZ no.: ACC 107, human lung carcinoma), and COS-7 (DSMZ no.: ACC 60, Afrikan green monkey kidney) cells were cultured in Dulbecco’s modified Eagle’s medium (DMEM) supplemented with 10% (v/v) fetal calf serum (FCS, Gibco), 100 μg/ml streptomycin, and 100 U/ml penicillin, HepG2 cells (DSMZ no.: ACC 180, human hepatocellular carcinoma) were cultured in RPMI 1640 medium supplemented with 10% (v/v) FCS, 1% (v/v) L-glutamine, streptomycin (100 μg/ml), and penicillin (100 U/ml). All cells were grown in a humidified atmosphere with 5% CO_2_ at 37°C, morphologically checked with an inverted microscope (DM IL LED, Leica Microsystems, Wetzlar, Germany) and passaged 2–3 times weekly.

**TABLE 1:** Chemical compounds with 18 carbon atoms derived from the chemical lead 9NOA.

Name	Chemical name	Molecular formular	NO_2_ position	Double bound position	E/Z ratio	Structure
18.4.4	4-nitro-octadec-4-enoic acid	C_18_H_33_NO_4_	4	4/5	95:5	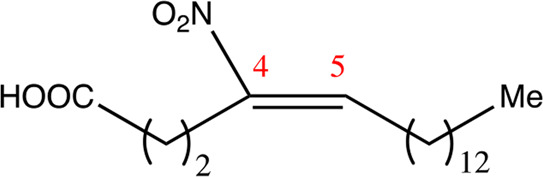
18.5.5	5-nitro-octadec-5-enoic acid	C_18_H_33_NO_4_	5	5/6	86:14	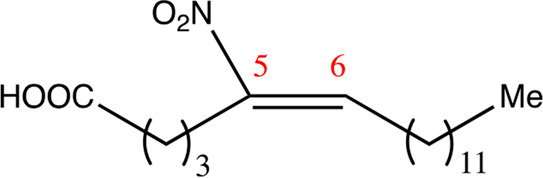
18.6.6	6-nitro-octadec-6-enoic acid	C_18_H_33_NO_4_	6	6/7	94:6	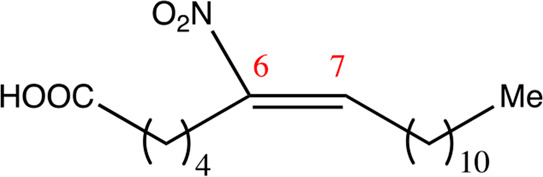
18.6.7	7-nitro-octadec-6-enoic acid	C_18_H_33_NO_4_	7	6/7	93:7	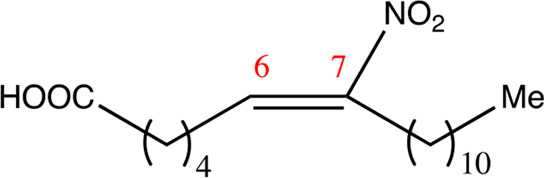
18.7.7	7-nitro-octadec-7-enoic acid	C_18_H_33_NO_4_	7	7/8	97:3	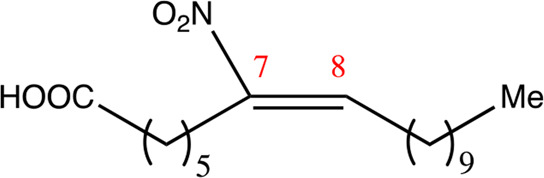
18.8.8	8-nitro-octadec-8-enoic acid	C_18_H_33_NO_4_	8	8/9	97:3	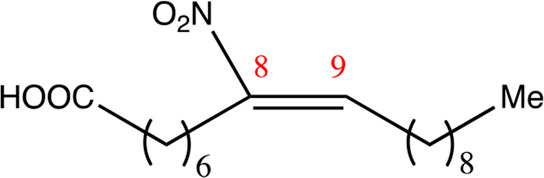
18.8.9	9-nitro-octadec-8-enoic acid	C_18_H_33_NO_4_	9	8/9	96:4	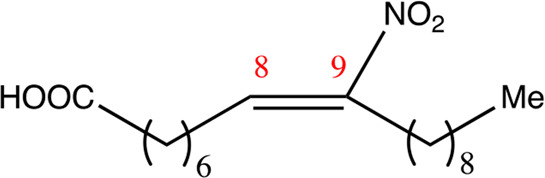
18.9.9	9-nitro-octadec-9-enoic acid	C_18_H_33_NO_4_	9	9/10	95:5	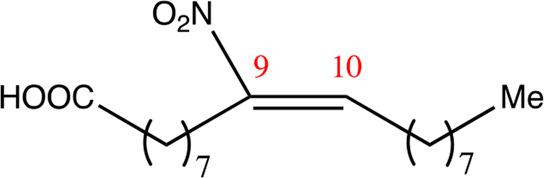
18.9.10	10-nitro-octadec-9-enoic acid	C_18_H_33_NO_4_	10	9/10	94:6	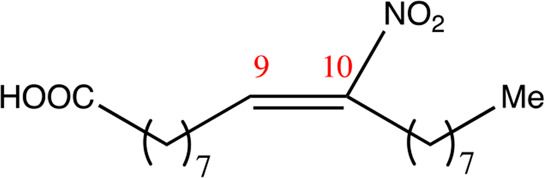
18.10.10	10-nitro-octadec-10-enoic acid	C_18_H_33_NO_4_	10	10/11	93:7	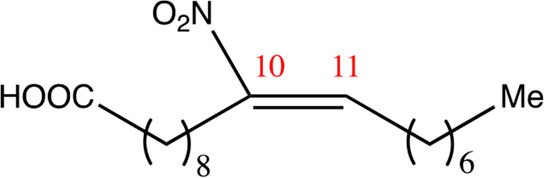
18.11.11	11-nitro-octadec-11-enoic acid	C_18_H_33_NO_4_	11	11/12	94:6	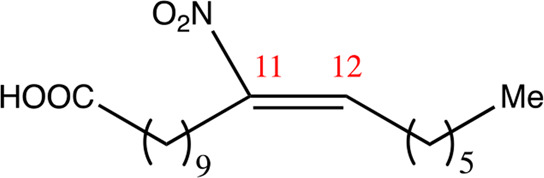

**TABLE 2:** Chemical compounds distinguished based on their numbers of carbon atoms derived from the chemical lead 9NOA.

Name	Chemical name	Molecular formular	NO_2_ position	Double bound position	E/Z ratio	Structure
12.9.9	9-nitro-dodec-9-enoic acid	C_12_H_21_NO_4_	9	9/10	93:7	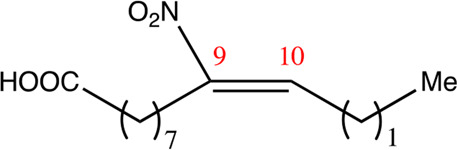
14.9.9	9-nitro-tetradec-9-enoic acid	C_14_H_25_NO_4_	9	9/10	94:6	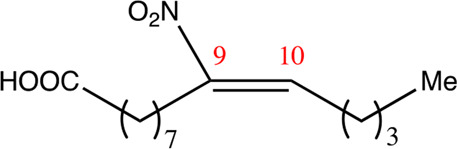
16.9.9	9-nitro-hexadec-9-enoic acid	C_16_H_29_NO_4_	9	9/10	93:7	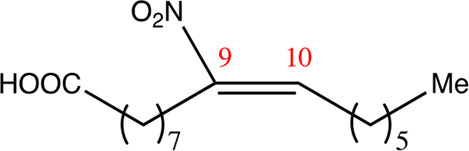
18.9.9	9-nitro-octadec-9-enoic acid	C_18_H_33_NO_4_	9	9/10	95:5	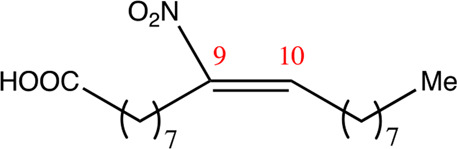
20.9.9	9-nitro-eicos-9-enoic acid	C_20_H_37_NO_4_	9	9/10	93:7	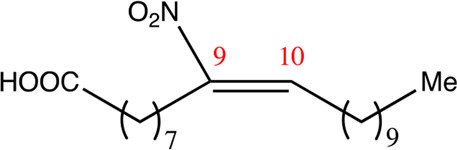
22.9.9	10-nitro-octadec-10-enoic acid	C_22_H_41_NO_4_	9	9/10	91:9	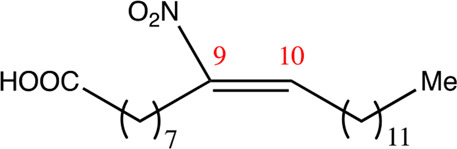

### 2.2 Firefly Luciferase Activity Assay

Recombinant firefly luciferase (Sigma-Aldrich, St. Louis, Missouri, United States) was diluted to 10^4^ U/mg in 1× PBS (phosphate buffered saline) and subsequently incubated with NFA or the substances of interest for 15 min at 37°C. Afterwards, 50 µl per reaction mix was transferred into a white 96-well plate, to which 50 µl/well Dual-Glo® (Promega, Madison, Wisconsin, United States) was added and incubated for another 10 min at 37°C. Luminescence was analyzed with a plate reader.

### 2.3 Enzyme-Linked Immunosorbent Assay

The Nrf-2 and NF-κB transcription factors in the nuclear extracts were detected using an Nrf-2 Transcription Assay Kit (Cayman Chemical, # 600590) and NF-κB (p65 and p50) Transcription Factor Assay Kit (Cayman Chemical, # 10007889 and # 10006912). Prior to assay performance, the nuclear extracts were generated using a Nuclear Extraction Kit (Cayman Chemical, # 10009277). The assay was performed according to the manufacturer’s instructions. For all kits, the nuclear extracts were incubated overnight at 4°C. Development was carried out for 15–20 min or until the wells turned medium to dark blue. Five minutes after the stop solution had been added, the absorbance was read at 450 nm using a plate reader.

### 2.4 Sodium Dodecyl Sulfate–Polyacrylamide Gel Electrophoresis and Western Blotting

HCT-116 and A549 cells were washed with 1× PBS, and cell extracts were generated using a Nuclear Extraction Kit (Cayman Chemical, #10009277) according to the manufacturer’s instructions, whereas whole-cell lysates were obtained using SDS lysis buffer (55.5 mM Tris-HCl pH 6.8, 2.2% SDS, 9% glycerol supplemented with protease (cOmplete® Protease Inhibitor Cocktail, Roche, Mannheim, Germany) and phosphatase (PhosStop® Easy Pack, Roche, Mannheim, Germany) inhibitors. Protein concentrations from the nuclear and cytosolic fractions were determined with a bicinchoninic acid (BCA) protein assay kit (Pierce™, Rockford, Illinois, United States). Cytoplasmic and nuclear extracts (100 µg each) and 30 µg whole-cell lysates were diluted in 5× Laemmli loading dye and denatured for 10 min at 95°C, loaded on a 10% SDS polyacrylamide gel, and separated by gel electrophoresis for 15 min at 80 V followed by 60 min at 120 V. The separated proteins were blotted onto a nitrocellulose membrane (Hybond-C Extra, Amersham Biosciences Ltd., UK) using the Mini Trans-Blot® module (Bio-Rad, Hercules, California, United States) for 90 min at 90 V. The membrane was blocked with Odyssey blocking buffer (Li-Cor Biosciences, Bad Homburg, Germany) or 5% milk powder in TBS-T (Tris Buffered Saline with 0.1% Tween) for 1 h at room temperature (RT). The membranes were incubated with the primary antibodies overnight at 4°C, and then with the secondary antibodies for 1 h at RT. The primary antibodies were diluted 1:1,000 and the secondary antibodies were diluted 1:10,000 to 1:20,000. The antibodies used were against Nrf-2 (Abcam, Cambridge, United Kingdom, #ab62352), COX-2 (Cayman Chemicals, Ann Arbor, Michigan, United States, #Cay160112-1), β-actin (Santa Cruz Biotechnology, Dallas, Texas, United States, #sc-47778), and lamin A/C (Cell Signaling, Danvers, Massachusetts, United States, #4777S). Upon detection, the membrane was washed three times with 1× PBS-T (Phosphate-Buffered Saline with 0.1% Tween) or TBS-T and finally once with 1× PBS or TBS before the protein–antibody interaction was visualized on an Odyssey Infrared Imaging System (Li-Cor Biosciences) at 700 nm or 800 nm depending on the fluorophore of the secondary antibody.

### 2.5 Recombinant sEH Activity Assay

sEH activity upon NFA treatment was investigated with an NFA Soluble Epoxide Hydrolase Inhibitor Screening Assay Kit (Cayman Chemicals, #10011671). The assay was performed according to the manufacturer’s instructions in a black 96-well microplate in duplicates. Following the addition of sEH substrate, fluorescence was measured in kinetic mode every 30 s for 30 min with a plate reader at excitation/emission wavelengths of 330/465 nm.

### 2.6 Cell-Based sEH Activity Assay

sEH activity in the cells was analyzed using a Soluble Epoxide Hydrolase Cell-Based Assay Kit (Cayman Chemical, #600090). HepG2 and COS-7 cells (5 × 10^4^) expressing highly active sEH were seeded in flat-bottom clear 96-well plates and allowed to become adherent for 3 h. Subsequently, fresh medium was added, and the cells were incubated with 10 µM NFA as well as 10 µM AUDA for 24 h at 37°C. Following incubation, the cells were processed according to the manufacturer’s instructions. The assay uses an internal signal subtraction to minimize potential errors generated by sEH-independent product formation due esterase mediated hydrolysis. The fluorescence intensity of the samples was measured in a plate reader at excitation/emission wavelengths of 330/465 nm sEH activity was calculated by the 6-methoxy-2-naphthaldehyde standard curve.

### 2.7 Isolation of Polymorphonuclear Leucocytes From Buffy Coats

Buffy coats from different donors of different blood types were commercially obtained from DRK Blutspendedienst Baden-Württemberg Institute Frankfurt. Donors have given their written consent. To avoid initial blood clotting only buffy coats from one donor or several donors with the same blood type were combined and diluted with 1× PBS mixed with 5% dextran solution and allowed to stand for 30 min at RT to separate the erythrocytes. Following separation, the supernatant was transferred very gently to new 50-ml tubes containing lymphocyte separation medium (LSM) and centrifuged for 10 min at 800 ×*g* at RT with the centrifuge brake turned off. Afterwards, the supernatant was decanted carefully to discard the platelets and monocytes. The PMNL were washed with 1× PBS before the residual erythrocytes were lysed in two cycles: the cell pellet was resuspended with 10 ml H_2_O and vortexed for 45 s, and lysis was stopped with 40 ml 1 × PBS. After 10-min centrifugation at 200 ×*g* at RT, the cycle was repeated once. The supernatant was aspirated, the cell pellet resuspended in 5 ml PBS/glucose and initially separated PMNL preparations with different blood types were finally pooled to gain a sufficient number of cells.

### 2.8 Lipoxygenase Activity Assay

5-LO activity in intact cells was assayed by measuring LTB_4_ (leukotriene B_4_) and 5-HETE (5-hydroxyeicosatetraenoic acid) levels in the PMNL supernatants. Since PMNL preparations (purity >95%) contain eosinophils expressing 15-LO-1 as well as platelets, we also analyzed the effects of NFA on the concomitant formation of 15-(S)-ETE and 12-(S)-HETE. Notably, 12(S)-HETE formation should mainly derive from platelet impurities within PMNL but small amounts may also derive from 15-LO-1. Recombinant 5-LO enzyme activity was assessed by the determination of 5-HETE levels representing a direct product assay. In the cell-based LO activity assays, 5 × 10^6^ cells per ml PBS/glucose were transferred to 5 ml glass tubes containing 1 mM CaCl_2_ and stored on ice. Thereafter, the cells were preincubated with NFA and with BWA4C (1 µM), U73122 (10 µM), oleic acid (OA), and vehicle control (0.1% DMSO) in a water bath at 37°C for 15 min. Subsequently, the cells were stimulated with calcium ionophore A23187 (2.5 µM) and arachidonic acid (AA, 20 µM) for an additional 10 min at 37°C. Recombinant 5-LO activity assays were carried out using 3 µg r5LO-wt enzyme. The enzyme was diluted in PBS/EDTA/ATP, transferred into 5-ml glass tubes, and stored on ice. Treatment with NFA and the controls OA, BWA4C, U73122, and vehicle control was carried out for 15 min on ice. The samples were then pre-heated at 37°C in a water bath and subsequently stimulated with 1 mM CaCl_2_ and 20 µM AA for 10 min at 37°C. The reaction was stopped by placing the glass tubes on ice. The supernatants were transferred to 1.5 ml reaction tubes and stored at −80°C before LC-MS (liquid chromatography and mass spectrometry) analysis of LTB_4_ and HETEs as described in 2.6 at the Institute of Pharmacology, Goethe University, University Hospital, Germany.

### 2.9 Analysis of Eicosanoids

#### 2.9.1 liquid phase extraction and LC-MS/MS

The lipid mediators LTB_4_, hydroxyeicosatetraenoic acids, 5(S)-HETE, 12(S)-HETE, 15(S)-HETE and 20-HETE were analyzed using liquid chromatography tandem-mass spectroscopy (LC-MS/MS). The LC-MS/MS system consisted of a 5500 QTrap mass spectrometer (Sciex, Darmstadt, Germany), operating in negative ESI mode, an Agilent 1200 HPLC system (Agilent, Waldbronn, Germany) and an HTC Pal autosampler (Chromtech, Idstein, Germany).

Sample extraction of LTB_4_ and HETEs were performed using liquid-liquid extraction: 200 µl of the sample were gently mixed with 20 µl of methanol and 20 µl of internal standard solution and extracted twice with 600 µl ethyl acetate. Samples for standard curve and quality control were prepared similarly: 200 µl PBS, 20 µl of standard solution and 20 µl internal standard solution were mixed and extracted with ethyl acetate. Working solutions of all analytes were prepared in methanol containing 0.1% BHT. The calibration standards were prepared by further dilution of the working standards.

The organic phase was removed at 45°C under a gentle stream of nitrogen. The residues were reconstituted in 50 µl of methanol:water:BHT (50:50:10^−4^, v/v/v) prior to injection into the LC-MS/MS system. Chromatographic separation was achieved using a Gemini NX C18 column (150 mm × 2 mm ID, 5 µm, Phenomenex, Aschaffenburg, Germany) with a precolumn of the same material. A linear gradient was employed at a flow rate of 0.5 ml/min and a total run time of 17.5 min. Mobile phases were A water:ammonia (100:0.05, v/v) and B acetonitrile:ammonia (100:0.05, v/v). The gradient started at 85% A, changed to 10% A within 12 min, held for 1 min, shifted back to 85% A in 0.5 min following 3.5 min equilibration.

All data were acquired using Analyst software v1.6.2 and quantitation was performed by MultiQuant software v3.0 (both Sciex, Darmstadt, Germany) using the internal standard method (isotope-dilution mass spectrometry). Calibration curves were calculated by linear regression with 1/x.

#### 2.9.2 Solid Phase Extraction and HPLC

For experiments shown in Table 4 samples after treatment were supplemented with 500 µl PBS, 30 µl HCl (1 N), and 200 ng internal standard prostaglandin B_1_. Those samples containing intact cells were centrifuged for 10 min (870 g, room temperature). Supplemented samples were then applied to RP18 solid phase Clean-Up extraction columns (United Chemical Technologies, Bristol, PA, United States), which were preconditioned with 1 ml methanol and 1 ml H2O. Columns were subsequently washed with 1 ml H2O and 1 ml methanol (25%; v/v), and 5-LO products were eluted using 300 µl methanol (100%). The eluate was diluted with 125 µl H_2_O and analyzed by reverse-phase HPLC as previously described (Steinhilber et al., 1989). Nova-PakC18 column (5 × 100 mm, 4 µm particle size; Waters, Eschborn, Germany) was used as a stationary phase in combination with methanol/water/TFA (72/28/0.007%; v/v/v) as the mobile phase. Unless otherwise stated, 5-LO metabolites 6-trans-LTB4, 12-epi-6-trans-LTB4, LTB4, and 5-H(p)ETE were quantified.

### 2.10 Recombinant COX-2 Activity Assay

The effects of NFA on COX-2 activity were analyzed using a recombinant COX-2 activity assay using a COX Fluorescent Inhibitor Screening Assay Kit (Cayman Chemicals, #700100). The assay was performed according to the manufacturer’s instructions in a black 96-well microplate. Human COX-2 was pre-incubated with NFA and control inhibitors for 30 min at RT following the addition of ADHP (10-acetyl-3,5-dihydroxyphenoxazine) and AA. The mixture was incubated again for 2 min at RT, and the fluorescence was measured at excitation/emission wavelengths of 535/587 nm.

### 2.11 Cell-Based COX-2 Activity Assay

The COX-2 inhibitory effects of NFA were investigated using a cell-based COX-2 activity assay performed using a Cyclooxygenase (COX) Activity Assay Kit (Fluorometric) from Abcam (Cambridge, UK; #ab204699). A549 cells (3 × 10^6^) were seeded in 10-cm dishes and allowed to become adherent for 3 h. The cells were stimulated with 1 ng/ml IL-1β and subsequently treated with 10 µM NFA or 5 µM celecoxib for 24 h. Then, cells were detached with a cell scraper and washed twice with 1× PBS before being resuspended in 100 µl lysis buffer (1× PBS containing 1% NP-40). The cells were incubated for 30 min with shaking on ice. After 3-min centrifugation at 12,000 ×*g* at 4°C, 20 µl supernatant was used for the COX activity assay. The assay was performed according to the manufacturer’s instructions in a white 96-well microplate with a clear bottom in duplicate. Fluorescence was measured at excitation/emission wavelengths of 535/587 nm in kinetic mode every 60 s for 30 min using a plate reader. COX-2 activity was normalized to protein concentration.

### 2.12 WST-1 Cell Viability Assay

Cell viability was analyzed with the WST-1 assay based on the cleavage of the stable tetrazolium salt WST-1 by the mitochondrial reductase system. Cells were seeded in the appropriate densities in a clear 96-well plate and settled overnight at 37°C in 5% CO_2_. The next day, the cells were treated with NFA or the substances of interest (≤0.1% dimethyl sulfoxide [DMSO]) and incubated for 24 or 48 h at 37°C in 5% CO_2_. Cell viability was analyzed by adding 10 µl WST-1 solution (Roche, Basel, Switzerland) and subsequently incubating the cells at 37°C for another 30–40 min. Absorbance was measured at 450 nm with a control wavelength at 620 nm using a plate reader.

### 2.13 Statistical Analysis

All data are presented as mean ± SD. GraphPad^®^ Prism version 7.00 was used for the statistical analysis. The tests performed are indicated in the figure legends. The sigmoidal dose-response curve fitting model “log(inhibitor) vs. normalized response-variable slope” was used to calculate the median inhibitory concentration values (IC_50_).

## 3 Results

### 3.1 Characterization of Synthesized NFA

NFA derivatives were synthesized and showed >95% purity, as described previously (Hock et al., 2017). The compounds were structurally analyzed by ^1^H-NMR and ^13^C-NMR. Five NFA derivatives with different chain lengths and 10 derivatives with nitroalkene groups located at positions 4–11 were obtained, and their cytoprotective, anti-inflammatory, and anti-tumorigenic activity were analyzed. Their structures are illustrated in Tables 1, 2.

### 3.2 Inhibition of TNFα-Induced NF-κB Activity by NFA

**FIGURE 1:**
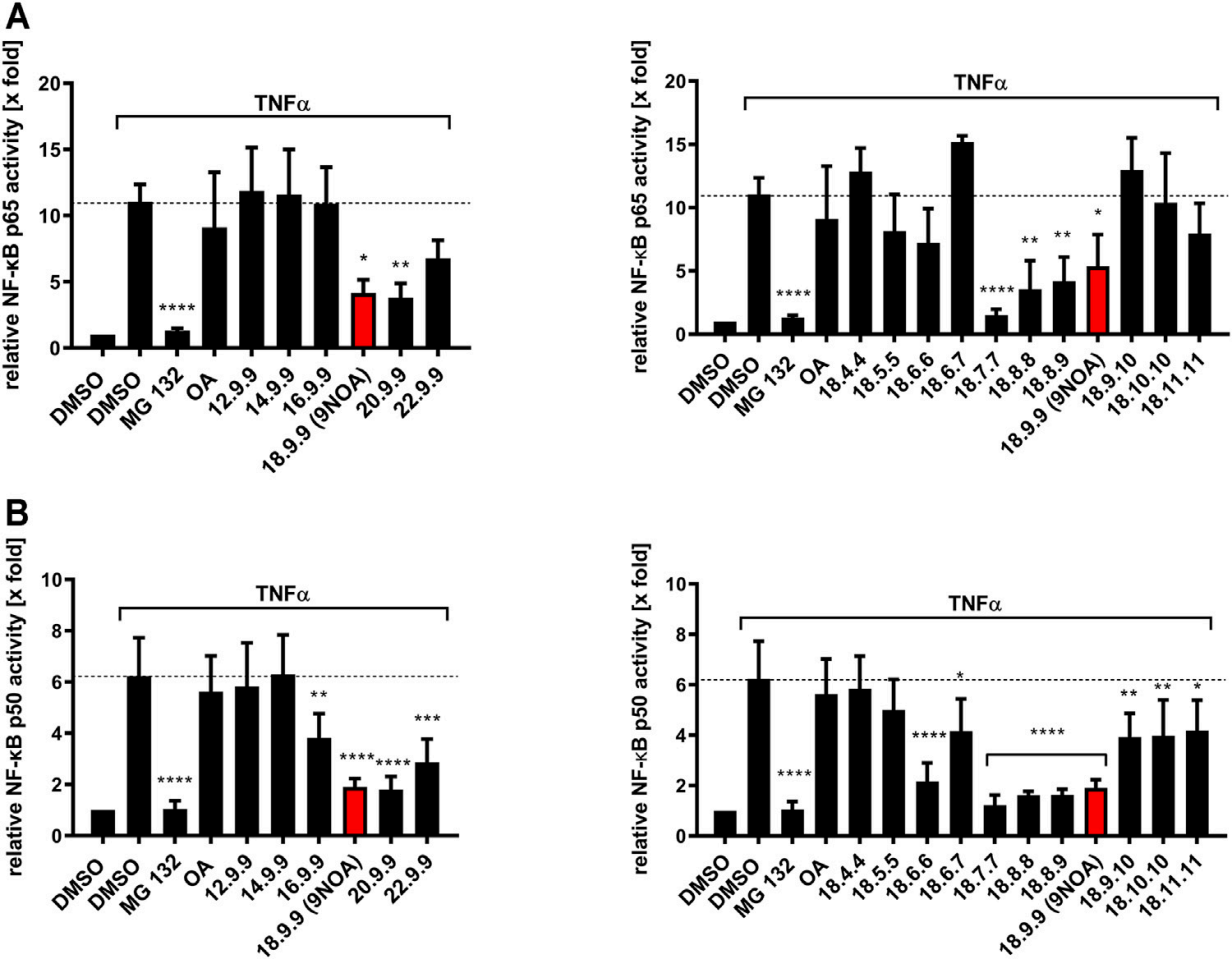
Effects of NFA on the NF-*κ*B p65 and p50 signaling pathway in colorectal cancer cells. HCT-116 cells were treated with 10 µM NFA and OA for 14 h; subsequently, NF-*κ*B signaling was induced by stimulation with 10 ng/ml TNF-α for an additional 4 h. Incubation for 4 h with the proteasome inhibitor MG132 (1 µM) served as the positive control. NF-*κ*B p65 **(A)** and p50 **(B)** inhibition by NFA with different carbon chain lengths and Michael acceptor positions were analyzed via ELISA. Values are normalized to protein concentration and unstimulated vehicle control. Data are represented as mean ± SD of (A) n = 3 (DMSO, 16.9.9, 9NOA control of right panel, 18.4.4, 18.6.7, and 18.9.10), *n* = 4 (all other derivatives) and **(B)** *n* = 5. Statistical significance was calculated by one-way analysis of variance (ANOVA) with Bonferroni post-test. *p ≤ 0.03, **p ≤ 0.0021, ***p ≤ 0.0002, ****p ≤ 0.0001 were considered significant versus TNF-α treatment. OA, oleic acid; NFA, nitro-fatty acids; TNF-α, tumor necrosis factor α; NF-κB, nuclear factor kappa B.

First, the effects of the NFA derivatives on TNFα-induced NF-κB activity were investigated. The proteasome inhibitor MG132 (Lee and Goldberg, 1998) was used as a positive control, as proteasomal inhibition leads to stabilization of I-κB and thus inhibits the activation of the NF-κB signaling pathway. We used a MG132 incubation period of 4 h as longer incubations have been described to activate NF-κB signaling (Lee et al., 2013). 1 µM 9NOA could significantly inhibit the recombinant firefly luciferase by more than 50%, while 10 µM 9NOA lead to complete inhibition of the enzyme. Similar effects were observed for the derivatives with differing Michael acceptor positions and with NFA with chain lengths of 20 and 22 carbon atoms, rendering the luciferase-based assays inapplicable for use with NFA (Supplymentary Figure S1) due to potential false-positive results. Alternatively, NFA inhibition of NF-κB activity was conducted using a p65-and p50-specific ELISA method after 14 h preincubation with the different NFA derivatives. We used this incubation period as preliminary experiments demonstrated only weak NF-κB inhibition at shorter preincubation periods and relevant cytotoxic effects at periods longer than 18 h. Notably, cytotoxic effects may be a consequence of NFA-mediated inhibition of NF-κB signaling and explain their anti-tumorigenic effects (Woodcock et al., 2018). 9NOA as the reference structure led to 50% inhibition of NF-κB p65 signaling (Figure 1A) and approximately 60% inhibition of NF-κB p50 signaling (Figure 1B), whereas OA did not affect NF-κB signaling (Figure 1) at 10 µM. NFA derivatives 18.7.7, 18.8.8, 18.8.9, and 18.9.9 were the most potent NF-κB p65 inhibitors at 10 µM concentration with 50% inhibition for 18.8.8, 18.8.9, and 18.9.9, and derivative 18.7.7 displaying the strongest inhibitory efficacy of almost 90%, which was similar to that of MG132 (Figure 1A). The other derivatives with a Michael acceptor position at 18.6.7 or closer to the carboxyl group or with a position of 18.9.10 or farther failed to suppress p65 NF-κB signaling. Similar to p65, NFA derivatives 18.7.7, 18.8.8, 18.8.9, and 18.9.9 were the most effective p50 inhibitors (Figure 1B). Moreover, all remaining NFA derivatives, except 18.4.4 and 18.5.5, produced significant albeit weaker NF-κB p50 inhibition. Interestingly, shortening the chain length to ≤16 carbon atoms led to the loss of any NF-κB-inhibitory activity, whereas elongating the chain length to 20 carbon atoms did not affect the inhibitory activity. However, moderately impaired inhibitory activity was seen with derivative 22.9.9 (Figure 1).

### 3.3 Cytoprotective Effects of NFA

**FIGURE 2:**
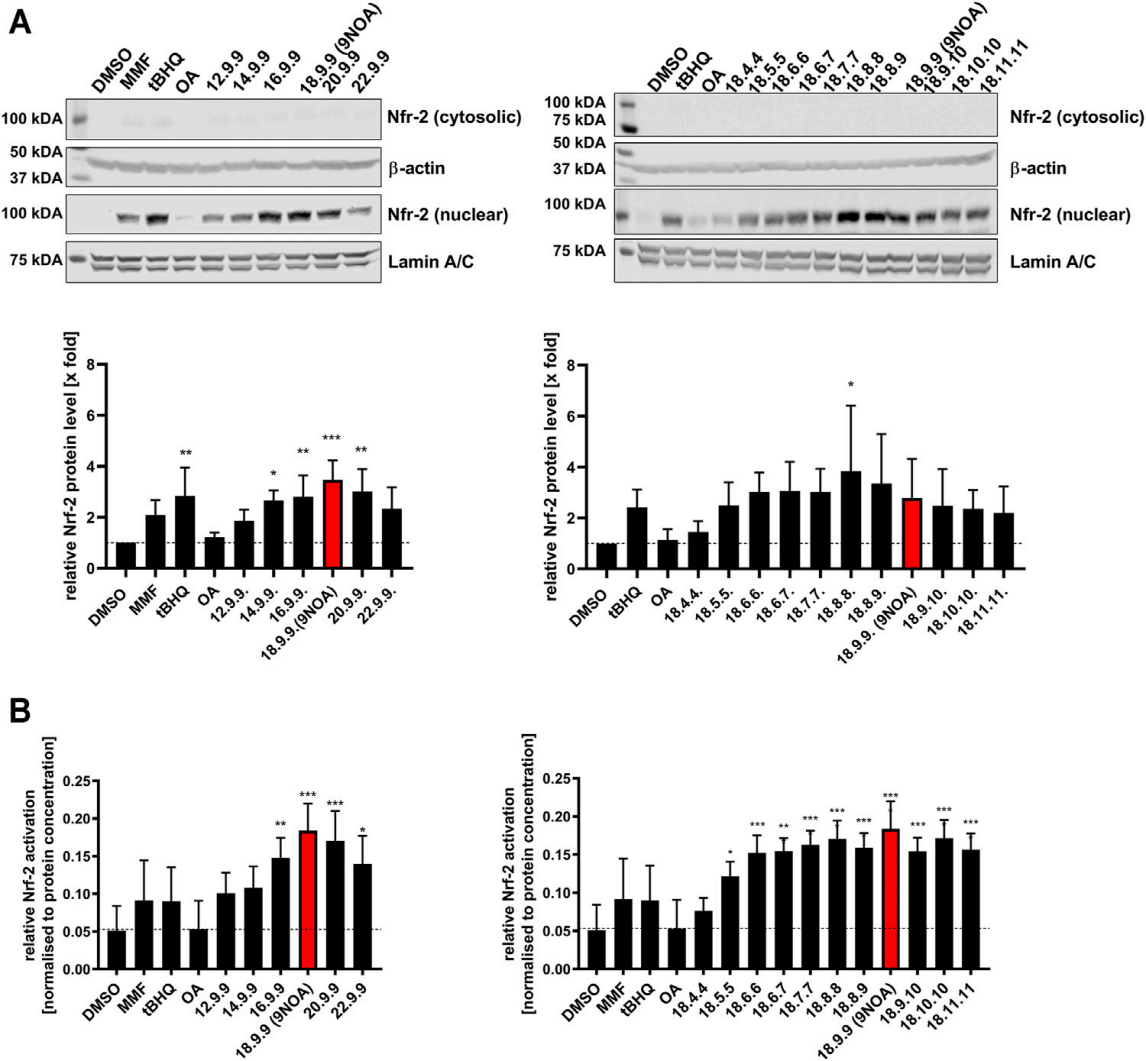
Effects of NFA on Nrf-2 expression in colorectal cancer cells. HCT-116 cells were treated with 10 µM NFA and tBHQ and/or MMF for 4 h, and Nrf-2 levels were analyzed by western blotting and ELISA. **(A)** Representative western blots of Nrf-2 levels from HCT-116 cells treated with NFA of different carbon chain lengths and different Michael acceptor positions (upper panel). Subcellular fractionation was performed, yielding cytosolic and nuclear extracts. ß-actin and Lamin A/C were used as respective loading controls (*n* = 4 experiments). Densitometric analysis of Nrf-2 protein levels is shown in the panel below. Cytosolic Nrf-2 protein levels were normalized against ß-actin and nuclear Nrf-2 levels against Lamin A/C. **(B)** Nrf-2 expression levels were evaluated with an ELISA after incubation with NFA with different carbon chain lengths and Michael acceptor positions. Values are normalized to protein concentration. Data are represented as mean ± SD of *n* = 4. Statistical analysis was performed using one-way ANOVA with Bonferroni post-test. *p ≤ 0.03, **p ≤ 0.0021, ***p ≤ 0.0002, ****p ≤ 0.0001 are considered significant. Nrf-2, nuclear factor erythroid 2-related factor; NFA, nitro-fatty acids; tBHQ, tert-butylhydroquinone; MMF, monomethyl fumarate.

NFA induce Nrf-2 signaling by the nitroalkylation of cysteines in Keap1 (Cole et al., 2009; Kansanen et al., 2009; Tsujita et al., 2011). As an alternative to determining Nrf-2 activity by firefly luciferase activity which is impaired due to the interaction of NFAs with luciferase enzyme activity (Supplementary Figure S1), Nrf-2 activation by NFA was analyzed using Western blotting and ELISA. We used an incubation period of 4 h as preliminary experiments revealed the strongest induction of Nrf-2 signaling by NFA after this incubation period (data not shown). The controls MMF (monomethyl fumarate) and tBHQ (tert-butylhydroquinone), two antioxidants altering Nrf-2 activity (Cho et al., 2015; Turley et al., 2015; Satoh and Lipton, 2017), led to an increase in Nrf-2 protein levels after 4-h incubation at 10 µM (Figure 2). All NFA derivatives induced Nrf-2 in HCT-116 cells after 4-h incubation, as observed in Western blot (Figure 2A) and ELISA (Figure 2B) experiments. Compared to MMF and tBHQ, NFA induction was equivalent, or in the majority of samples, increased. NFA with chain lengths of >16 carbon atoms significantly induced Nrf-2 gene expression (Figure 2B). All derivatives with a Michael acceptor position of ≥6 led to significant and approximately 3-fold induction of Nrf-2 signaling (Figure 2B). As a further negative control, OA did not affect Nrf-2 gene expression.

### 3.4 NFA Inhibition of sEH

**FIGURE 3:**
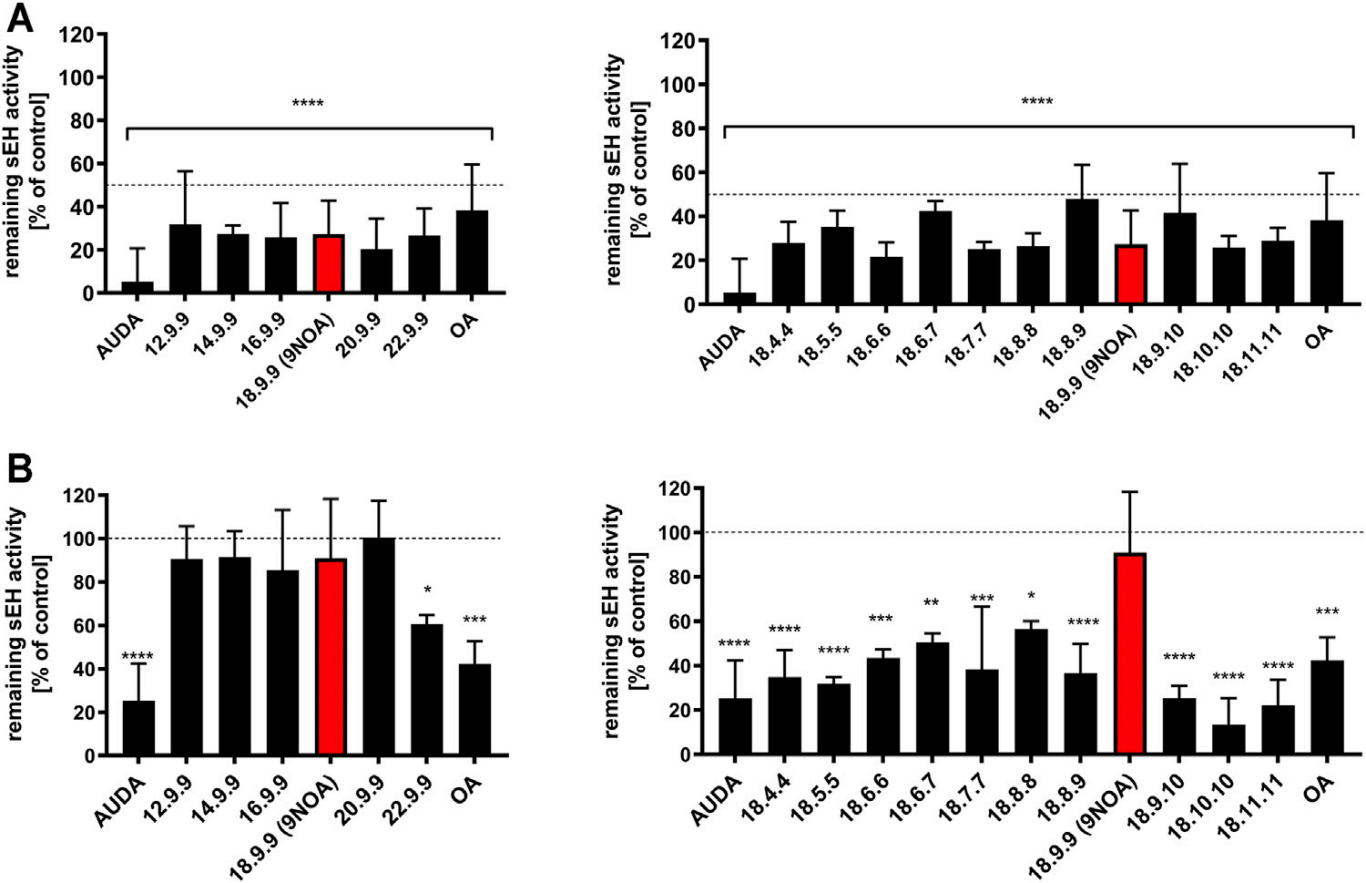
Effects of NFA on sEH activity. **(A)** Human recombinant sEH was incubated with 100 nM OA and NFA of different carbon chain lengths and Michael acceptor positions. AUDA (10 nM) served as the positive control. sEH activity was measured by the release of 6-methoxy-2-naphthaldehyde from epoxidized PHOME. Data are the mean ± SEM of *n* = 3–7. **(B)** sEH activity of HepG2 cells treated with NFA and AUDA (10 µM) for 24 h. The cells were lysed, and hydrolase activity was measured by Epoxy Fluor 7 lysis. Data have been normalized against DMSO control and therefore are presented as the percent of the control mean ± SEM, *n* = 2–3. Statistical significance was calculated by one-way ANOVA with Bonferroni post-test and multiple comparisons against the DMSO control. As indicated all data are significant at ****p ≤ 0.0001. *p ≤ 0.03, **p ≤ 0.0021, ***p ≤ 0.0002 are also considered significant. sEH, soluble epoxide hydrolase; OA, oleic acid; NFA, nitro-fatty acids; PHOME, 3-phenyl-cyano(6-methoxy-2-naphthalenyl)methyl ester-2-oxiraneacetic acid.

Via nitroalkylation of sEH cysteine within the catalytic center, NFA efficiently modulate EET levels and therefore induce vasodilatation (Charles et al., 2011; Charles et al., 2014). The effects of NFA on the recombinant sEH using 100 nM concentration differed from the observed results in intact cells using 10 µM of NFA (Figure 3). The sEH inhibitor AUDA (Zhang et al., 2015) caused 80–95% inhibition at concentrations of 10 nM for the recombinant, and 10 µM for the cellular sEH. While NFA with chain lengths of 12 up to 22 carbon atoms significantly inhibited recombinant sEH by >50% at (Figure 3A, left panel), the effect on sEH in intact cells (HepG2 and COS-7) was much weaker (Figure 3B; Supplymentary Figure S2). Only NFA derivative 22.9.9 caused a significant reduction in sEH activity in HepG2 cells by approximately 40%. When NFA with different Michael acceptor moiety positions were compared, all NFA derivatives showed significant enzyme inhibition at a concentration of 100 nM within the recombinant system (Figure 3A, right panel). In intact HepG2 cells, sEH activity was significantly reduced by all NFA derivatives, except the 18.9.9 derivative (Figure 3B, right panel). Shifting the Michael acceptor towards the ω-chain end in NFA 18.9.10, 18.10.10, and 18.11.11 led to the most efficient enzyme inhibition (Figure 3B, right panel). Notably, OA demonstrated an unexpected and strong suppressive effect on sEH activity in the recombinant system (Figure 3A) and in HepG2 (Figure 3B) and COS-7 cells (Supplymentary Figure S2), bringing into question the role of the Michael acceptor moiety in sEH inhibition.

### 3.5 Effects of NFA on LO-Derived Lipid Mediators

5-LO, the key enzyme in leukotriene biosynthesis, catalyzes the first steps in leukotriene formation from AA. Oxidation of AA by 5-LO leads to the formation of 5-hydroperoxyeicosatetraenoic acid (5-HPETE), which can further be converted to LTA_4_ or reduced to 5-HETE. In the next step, LTA_4_ is hydrolyzed by LTA_4_ hydrolase to produce LTB_4._ Moreover, 12- and 15-LO catalyze the formation of 12- and 15-H(P)ETE [12- and 15-hydro(per)oxyeicosatetraenoic acid]. Nitroalkylation of 5-LO cysteines 416 and 418 by NFA leads to enzyme inhibition and beneficial anti-inflammatory effects, whereas no impact has been observed on 12- and 15-LO so far (Awwad et al., 2014). 5-LO product formation of purified PMNL was triggered by incubation with 2.5 µM of the calcium ionophore A23187 in the presence of 20 µM AA. The validity of our PMNL assay was controlled using the phospholipase C inhibitor U73122 (10 µM) and 5-lipoxygenase inhibitor BWA4C (1 µM), leading to >90% inhibition of 5-LO product formation (data not shown). Table 3 shows that shortening NFA down to 12 carbon atoms strongly impaired their effect on LTB_4_ and 5-HETE production in intact PMNL. Increasing the chain length to 22 atoms moderately increased the 5-LO inhibitory potency in comparison to 9NOA. All NFA derivatives failed to suppress 12-HETE production, and 15-HETE production was inhibited by NFA derivatives >16 carbon atoms only at concentrations of >10 µM. By contrast, altered Michael acceptor position had no significant impact on LTB_4_ and 5-HETE production (Tables 3, 4), with NFA 18.7.7 slightly increasing the inhibition of LTB_4_ and 5-HETE formation in intact PMNL compared to 9NOA (Table 3). The IC_50_ could be slightly reduced from 4.8 ± 1.8 µM (9NOA) to 4.3 ± 1.1 µM (18.7.7) for LTB_4_ and from 6.1µM ± 1.1 (9NOA) to 4.9 ± 1.1 µM (18.7.7) for 5-HETE synthesis (Tables 3, 4). Whereas 18.9.9 (9NOA) suppressed 15-LO product formation in PMNL only at rather high concentrations of >10 µM (IC_50_ = 20.4 ± 1.1 µM), shifting the Michael acceptor moiety towards the carboxyl group increased the 15-LO-inhibitory potency, reaching an IC_50_ of 7.6 ± 1.1 µM for the 18.4.4 derivative. Shifting the nitro group from position 9 to 10, or rather, towards position 11 (18.11.11), led to a decreased impact on 15-LO product formation (Table 3). NFA 20.9.9 and 22.9.9 showed the highest inhibitory potency among all NFA derivatives tested and have the highest probability to compete with AA for the binding to 5-LO. Therefore, 5-LO product formation of purified PMNL after treatment with A23187 was determined in absence of AA which should strongly increase the 5-LO-inhibitory potency of competitive inhibitors. However, IC_50_ values were similar under these conditions strengthening the hypothesis of NFA as presumably non-competitive 5-LO inhibitors. Using a WST-1 viability assay we assessed possible cytotoxic effects of the strongest 5-LO-inhibitory NFA derivatives in PMNL including 18.4.4, 18.5.5, 18.6.6, 18.7.7, 18.8.8, 18.8.9, 18.9.9, 18.9.10, 18.10.10, 20.9.9, and 22.9.9. However, treatment of PMNL with 20 µM of these NFA derivatives for 2 h failed to induce significant cytotoxic effects (data not shown).

**TABLE 3:** IC_50_ values of inhibitory effects on LTB_4_ and eicosanoid-producing LOs after NFA treatment of intact PMNL and r5-LO in presence of 20 µM AA. Data are the mean of at least three independent experiments, *n* = 3. Total LO product formation in presence of 20 µM AA (5 × 10^6^ cells) was 89.5 ± 30.5 ng/ml (LTB_4_), 527.3.2 ± 278.7 ng/ml (5-HETE)), 156.5 ± 53.5 ng/ml (12-HETE) and 186.7 ± 94 ng/ml (15-HETE). Total product formation of recombinant 5-LO was 104.0 ± 54.7 ng/ml (5-HETE).

Compound	PMNL (20 µM AA)				r5LO-wt
	LTB_4_	5-HETE	12-HETE	15-HETE	5-HETE
		IC_50_ [µM]			
**OA**	n.i.	n.i.	n.i.	n.i.	n.i.
**12.9.9**	n.i.	n.i.	n.i.	n.i.	75.5 ± 8.1
**14.9.9**	n.i.	n.i.	n.i.	n.i.	17.9 ± 1.6
**16.9.9**	12 ± 1.1	10.8 ± 1.1	n.i.	n.i.	3.3 ± 1.2
**18.9.9**	**4.8 ± 1.8**	**6.1 ± 1.1**	**n.i.**	**20.4 ± 1.1**	**1.1 ± 1.2**
**20.9.9**	4.2 ± 1.1	4.7 ± 1.1	n.i.	21.2 ± 1.1	0.9 ± 1.3
**22.9.9**	2.9 ± 1.1	3.3 ± 1.1	n.i.	21.7 ± 1.3	0.3 ± 1.2
**18.4.4**	10.6 ± 1.1	9.2 ± 1.2	n.i.	7.7 ± 1.2	0.4 ± 1.2
**18.5.5**	9.0 ± 1.1	7.5 ± 1.1	n.i.	8.9 ± 1.2	0.4 ± 1.1
**18.6.6**	7.7 ± 1.1	9.1 ± 1.2	n.i.	14.8 ± 1.1	1.0 ± 1.2
**18.6.7**	8.3 ± 1.1	11.2 ± 1.2	n.i.	17.3 ± 1.1	1.1 ± 1.2
**18.7.7**	4.3 ± 1.1	4.9 ± 1.1	n.i.	7.6 ± 1.1	0.6 ± 1.3
**18.8.8**	7.9 ± 1.1	6.6 ± 1.1	n.i.	16.0 ± 1.1	2.2 ± 1.2
**18.8.9**	5.7 ± 1.2	7.7 ± 1.2	n.i.	23.4 ± 1.3	1.9 ± 1.2
**18.9.9**	**4.8 ± 1.1**	**5.9 ± 1.1**	**n.i.**	**17.6 ± 1.1**	**1.1 ± 1.2**
**18.9.10**	5.0 ± 1.1	6.9 ± 1.1	n.i.	20.5 ± 1.3	1.6 ± 1.3
**18.10.10**	7.0 ± 1.1	6.0 ± 1.2	n.i.	19.6 ± 1.1	1.6 ± 1.3
**18.11.11**	5.9 ± 1.1	5.7 ± 1.1	n.i.	26 ± 1.3	0.8 ± 1.2

**TABLE 4:** IC_50_ values of inhibitory effects on LTB_4_ and eicosanoid-producing LOs after NFA treatment of intact PMNL in absence of AA. Data are the mean of at least three independent experiments, *n* = 3. LO product formation in absence of AA (5 × 10^6^ cells) was 106.2 ± 48.7 ng/ml (LTB_4_), 80.9 ± 10.7 ng/ml (5-HETE) and 62.4 ± 83.1 ng/ml (12-HETE). n.d. not determined.

Compound	PMNL (w/o AA)			
	LTB_4_	5-HETE	12-HETE	15-HETE
	IC_50_ [µM]			
**18.9.9**	**2.58 ± 1.4**	**2.34 ± 1.50**	**6.26 ± 0.823**	**n.d.**
**20.9.9**	2.05 ± 1.25	2.06 ± 1.34	15.49 ± 0.74	n.d
**22.9.9**	1.01 ± 1.4	1.04 ± 1.28	4.38 ± 1.00	n.d

### 3.6 NFA Inhibition of 5-LO

As 9NOA is a direct 5-LO inhibitor, we analyzed the effect of the NFA derivatives on recombinant 5-LO enzyme activity. As with intact PMNL, shortening the 18.9.9 derivative systematically impaired the potency of the NFA derivative to inhibit recombinant 5-LO, whereas increasing the chain length to 20 or 22 carbon atoms led to increased 5-LO inhibitory potency compared to 9NOA (Supplementary Figure S3). The long-chain NFA derivative 22.9.9 was most potent among all NFA derivatives tested and inhibited recombinant 5-LO activity with an IC_50_ of 0.3 ± 1.2 µM (Tables 3,4). Alteration of the Michael acceptor position hardly affected the effect of the derivatives on recombinant 5-LO enzyme activity (Supplementary Figure S3; Tables 3, 4). Accordingly, the IC_50_ of 1.1 ± 1.2 µM 9NOA was reduced to an IC_50_ of 0.4 ± 1.2 µM with derivative 18.4.4 (Tables 3, 4). OA only showed a slight inhibitory effect on the product formation by recombinant 5-LO at concentrations of >10 µM. 12- and 15-LO were not affected by OA (Supplementary Figure S4, Table 3, 4).

### 3.7 Effects of NFA on COX-2

**FIGURE 4:**
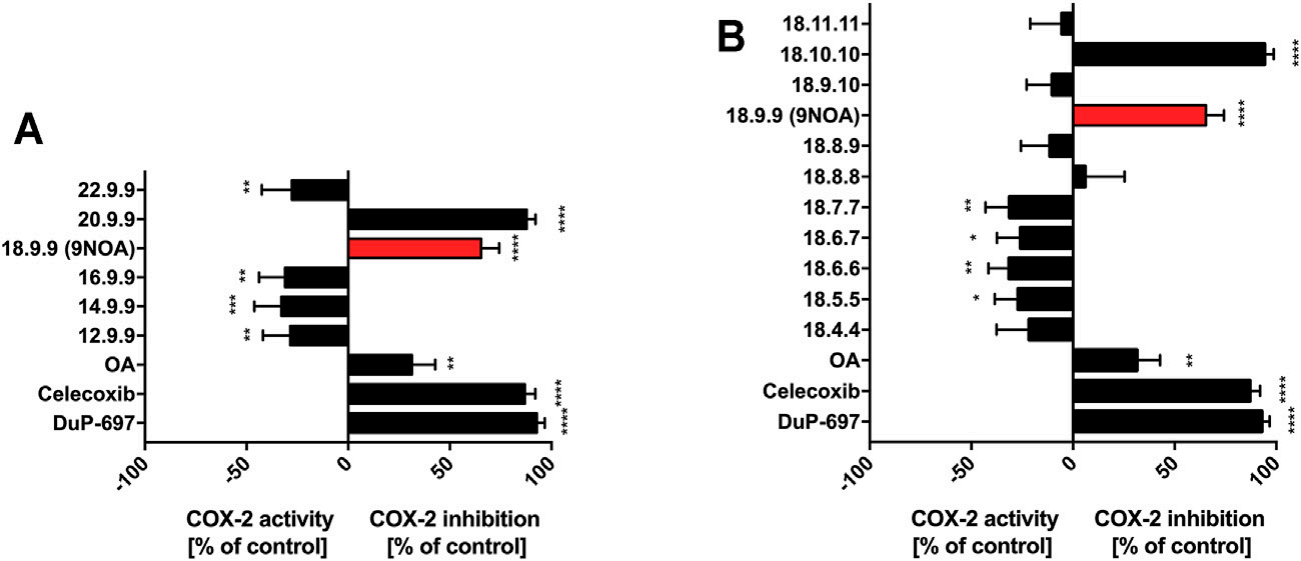
Effects of NFA on COX-2 peroxidase activity. Human recombinant COX-2 was incubated with 10 µM OA and NFA with different **(A)** carbon chain lengths and **(B)** Michael acceptor positions. Celecoxib (5 µM) and DuP-697 (3 µM) served as positive control. Peroxidase activity was measured by the appearance of TMPD. Data are presented as the percent of the control mean ± SD, *n* = 4. Statistical significance was calculated by one-way ANOVA with Bonferroni post-test. **p ≤ 0.0021, ***p ≤ 0.0002, ****p ≤ 0.0001 are considered significant. NFA, nitro-fatty acids; TMPD, N,N,N‘,N‘-tetramethyl-p-phenylenediamine; COX-2, cyclooxygenase 2; OA, oleic acid.

Prostaglandins play an important role in inflammation and pain, and their formation mainly relies on the activity of COX-1 and COX-2. The formation of prostaglandin G_2_ (PGG_2_) and the subsequent reduction to prostaglandin H_2_ (PGH_2_) is catalyzed by COX (van der Donk et al., 2002). Activation of the enzyme depends on COX peroxidase activity (Rouzer and Marnett, 2009). Moreover, the inhibition of peroxidase, but not oxygenase, activity could be observed for NFA regarding COX-2 (Trostchansky et al., 2011). Therefore, we analyzed the inhibition of COX-2 peroxidase activity by NFA derivatives using a recombinant and cell-based COX-2 peroxidase activity assay. COX-2 expression was further analyzed in western blot studies. The vast majority of NFA were unable to inhibit human recombinant COX-2, rather, they led to increased COX-2 activity (Figure 4). However, the structure–activity relationship did not follow clear systematics. At 10 µM, 9NOA (inhibition >65%) as well as derivative 20.9.9 (inhibition >85%) significantly suppressed the activity of recombinant COX-2. Surprisingly, further elongation of the carbon chain by two carbon atoms to yield derivative 22.9.9 rendered the derivative a weak COX-2 activator at 10 µM. This was also observed if the chain length was shortened to 16, 14, or 12 carbon atoms, converting these derivatives into weak COX-2 activators (Figure 4A). The COX-2 inhibitors celecoxib (5 µM) and DuP-697 (3 µM), used as controls, produced enzyme inhibition of >85% (Figure 4). OA only weakly affected COX-2 activity. Shifting the Michael acceptor moiety to position 9.10 and 11.11 produced weak COX-2- activators, whereas 10 µM NFA 18.10.10 inhibited COX-2 even more efficiently than NFA 18.9.9 (9NOA), reaching >90% inhibition (Figure 4B). All NFA derivatives with a Michael acceptor moiety at positions 4–7 showed increased enzyme activity (Figure 4B). In IL-1β–induced A549 lung carcinoma cells expressing highly active COX-2 as published previously (Petkova et al., 2004), no COX-2 inhibition by 10 µM 9NOA or derivative 20.9.9 could be observed as compared to the control OA (Supplymentary Figure S5A). In contrast, NFA derivative slightly 20.9.9 increased COX-2 product formation. Whereas derivative 18.10.10 produced slight COX-2 inhibition in IL-1β–stimulated A549 cells, derivatives 18.6.6 and 18.8.8 induced COX-2 activity (Supplementary Figure S5B). We also investigated the effects of 9NOA and the NFA derivatives on COX-2 expression in the presence and absence of IL-1β in A549 cells, as NFA-mediated changes in COX-2 protein levels might have contributed to the altered COX-2 activity levels observed in Supplemenatry Figure S5. In non-stimulated A549 cells, only NFA 18.7.7, and to a lesser extent, NFA 20.9.9 and 18.8.8, clearly increased COX-2 gene expression (Supplementary Figures 6A–D). In IL-1β–treated A549 cells, 10 µM NFA with ≥16 carbon atoms induced COX-2 gene expression (Supplementary Figures S6E,G). All NFA derivatives with differing Michael acceptor positions could induce COX-2 expression in IL-1β–stimulated A549 cells, with NFA 18.5.5, 18.7.7, and 18.8.8 showing the highest effects (Supplementary Figures 6F,H). The COX-2 inhibitor celecoxib (5 µM) also induced COX-2 in IL-1β–stimulated A549 cells (Supplementary Figures S6E–H), albeit the compound strongly inhibited COX-2 activity in the cellular system (Supplementary Figure S5).

### 3.8 Determination of the Cytotoxic Effects of NFA in Colorectal Cancer Cells

Besides targeting the NF-κB pathway, the anti-tumorigenic effects of NFA can be observed by a reduction of colorectal cancer cell viability. Previously, we demonstrated that NFA and few derivatives exert cytotoxic effects on colorectal cancer cells, including HCT-116 and HT-29 and HEK 293 cells, respectively, by inducing a caspase-dependent apoptosis pathway and cell cycle arrest, accompanied by mitochondrial dysfunction (Kühn et al., 2018). Again, the cytotoxic effects of NFA were determined in HCT-116 and HT-29 colorectal cancer cells in the present study. The well-recognized anti-tumorigenic agent thymoquinone suppressed HCT-116 and HT-29 cell viability effectively, whereas OA failed to induce cytotoxic effects (Table 5). HCT-116 cells were treated with increasing concentrations of NFA differing in chain length and Michael acceptor position for 24 h, and cell viability was determined using the WST-1 assay. Due to their prolonged cell division rate and their lack of sensitivity towards NFA (Kühn et al., 2018), HT-29 cells were treated for 48 h to gain improved NFA activity.

**TABLE 5:** IC_50_ values of cytotoxic effects of NFA in colorectal cancer cell lines. Cell viability was analyzed using the WST-1 assay. Data are the mean of at least four independent experiments, *n* = 4.

Compound	HCT-116 (24 h)	HT-29 (48 h)
	IC_50_ [µM]	IC_50_ [µM]
**OA**	n.i.	n.i.
**TQ**	4.2 ± 1.2	10.3 ± 1.1
**12.9.9**	23.1 ± 1.1	∼31.5 ± 1.5
**14.9.9**	16.8 ± 1.1	27.4 ± 1.0
**16.9.9**	10.5 ± 1.1	12.7 ± 1.1
**18.9.9**	**6.4 ± 1.2**	**8.8 ± 1.1**
**20.9.9**	5.4 ± 1.1	8.1 ± 1.1
**22.9.9**	10.6 ± 1.1	15.8 ± 1.1
**18.4.4**	18.6 ± 1.1	23.9 ± 1.1
**18.5.5**	11.6 ± 1.1	18.5 ± 1.1
**18.6.6**	7.6 ± 1.1	11.1 ± 1.1
**18.6.7**	8.1 ± 1.1	13.8 ± 1.1
**18.7.7**	2.7 ± 1.1	4.4 ± 1.1
**18.8.8**	2.1 ± 1.1	3.3 ± 1.1
**18.8.9**	4.6 ± 1.1	8.0 ± 1.1
**18.9.9**	**6.4 ± 1.2**	**8.8 ± 1.1**
**18.9.10**	13.5 ± 1.1	15.7 ± 1.1
**18.10.10**	13.6 ± 1.1	13.9 ± 1.1
**18.11.11**	16.0 ± 1.1	16.6 ± 1.1

n.i.: no inhibition observed.

NFA with carbon chain lengths of <18 carbon atoms produced clearly weaker cytotoxic effects in HCT-116 cells than 9NOA (IC_50_ = 6.4 ± 1.2 µM, Table 5). Accordingly, in HCT-116 cells, the IC_50_ values for NFA 12.9.9 and 14.9.9 were 23.1 ± 1.1 µM and 16.8 ± 1.1 µM, respectively, and thus were approximately 3–4-fold higher compared to 9NOA. NFA 16.9.9 and 22.9.9 showed similar cytotoxicity, with IC_50_ values of 10.5 ± 1.1 µM and 10.6 ± 1.1 µM, respectively. NFA 20.9.9 was slightly more potent than the reference compound 9NOA, with an IC_50_ of 5.4 ± 1.1 µM (Table 5). Similar dependencies of cytotoxic potency on chain length were observed in the HT-29 cells (Table 5). Regarding the dependency Michael acceptor position, NFA derivatives with Michael acceptor moiety at positions 7 and 8 were the most potent cytotoxic derivatives both in the HCT-116 and HT-29 cells (Table 5). Cytotoxic potencies of 18.7.7 and 18.8.8 were >2-fold higher compared to that of 9NOA (IC_50_ = 2.7 ± 1.1 and 2.1 ± 1.1 µM in HCT-116 and IC_50_ = 4.4 ± 1.1 and 3.3 ± 1.1 µM in HT-29 cells, respectively, Table 5). Furthermore, changing the double bond of 9NOA from position 9 to 8, yielding NFA 18.8.9, slightly increased cytotoxicity, with a greater impact on HCT-116 cells than HT-29 cells (Table 5).

## 4 Discussion

The present study was aimed at addressing the relationship between the chemical structure of NFA and their biological activity on distinct signaling pathways or selected target proteins. Our study was based on the hypothesis that changing the chain length or position of the Michael acceptor moiety of 9NOA might have a strong impact on the biological effects of NFA. Here, we show that changing these structural features made it possible to increase the potency of certain NFA derivatives on one target (pathway), whereas the effects on other targets (pathways) were either impaired or even lost (Supplementary Figure S7).

NF-κB signaling is among the first described direct NFA targets. NFA inhibit NF-κB signaling via IKKβ inhibition which results in reduced degradation of IKBα and the subsequent inhibition of p65/p50 translocation into the nucleus. Furthermore, nitroalkylation of the p65 and p50 subunits leads to reduced DNA-binding activity of these transcription factors (Khoo et al., 2018). NFA show a similar pattern for p65 and p50 NF-κB inhibition, as most of the analyzed NFA derivatives revealed equivalent efficacy targeting p65 and p50. We were able to demonstrate that the NFA chain length has a great impact on modulation of NF-κB signaling, as NFA with <18 carbon atoms showed clearly reduced suppressive effects compared to 9NOA (Figure 1). NFA with a Michael acceptor moiety between position 7 and 9 (NFA 18.7.7–18.9.9) were the most efficient NF-κB inhibitors, with a slightly greater impact on p50 than p65. The fact that the NFA p65 and p50 inhibition patterns appeared quite similar led to the assumption that their mode of action might not be p50-or p65-targeted, but more upstream of the NF-κB signaling pathway.

Nrf-2 activators induce the gene expression of antioxidants, leading to cellular protection against oxidative stress. Moreover, Nrf-2-inducing drugs have beneficial effects on diseases such as obesity, diabetes, arteriosclerosis, neurodegenerative diseases, and tumorigenesis (Joshi and A. Johnson, 2012; Magesh et al., 2012; Suzuki et al., 2013). NFA lead to the nitroalkylation of Keap1 cysteines 273 and 288, causing Nrf-2 accumulation in the nucleus and antioxidant gene expression (Kansanen et al., 2009; Tsujita et al., 2011; Khoo et al., 2018). Here, nearly all tested NFA derivatives showed stronger Nrf-2 induction than MMF and tBHQ. The strongest effects were seen with naturally occurring NFA 18.9.9, which might represent the optimal structure for inducing Nrf-2. Short-chain NFA with <16 carbon atoms displayed weaker induction of the Nrf-2 pathway. Nevertheless, due to beta-oxidation, short-chain NFA are produced either way due to intracellular fatty acid degeneration. In line with literature, induction of Nrf-2 by NFA occurred rapidly after 4 h which accords with the reported nitroalkylation of Keap1 and the subsequent rapid release of Nrf-2 from the Keap1-mediated degradation increasing Nrf-2 protein stability and causing rapid translocation to the nucleus (Zhang and Hannink, 2003).

By contrast, chain length seems to be of minor importance for cellular sEH inhibition, as only NFA 22.9.9 led to significant sEH inhibition in HepG2 cells. However, minor differences in Michael acceptor position in NFA 18.9.10, 18.10.10, and 18.11.11 rendered these molecules potent cellular sEH enzyme inhibitors. Notably, OA led to clear inhibition of sEH by more than 50% questioning the exclusive role of the Michael acceptor moiety in NFA-mediated inhibition of sEH. However, as can be seen with 18.9.9 and OA compared to 18.9.10, 18.10.10, changing the position of the Michael acceptor moiety can further increase the sEH-inhibitory potency suggesting that the nitroalkylation of sEH plays a role in the inhibition of cellular sEH by the sEH-inhibitory compounds. Nevertheless, the use of our NFA derivatives allowed more precise insights into the role of NFA in inhibition of sEH. Thus, our experiments question NFA derivatives as promising scaffold for the design of selective sEH inhibitors.

NFA directly inhibit 5-LO by nitroalkylating the regulatory cysteines 416 and 418. In agreement with our previous studies, we were able to demonstrate that NFA have no or only minor effects on 12-LO and 15-LO activity, respectively (Table 3). Interestingly, NFA with longer chain lengths were more potent 5-LO inhibitors than short-chain NFA (Tables 3, 4). As AA, containing 20 carbon atoms, is a natural substrate for 5-, 12-, and 15-LO, it is reasonable to speculate that substrate similarity might be the reason for the increased 5-LO inhibitory potency of NFA 20.9.9 and 22.9.9. However, changing the position of the Michael acceptor moiety only weakly affected 5-LO inhibition by NFA, with NFA 18.7.7 being the only inhibitor showing increased 5-LO inhibition. Surprisingly, the shift of the nitro group towards the carboxyl group led to strongly elevated 15-LO inhibitory activity, whereas displacement towards the ω-end further weakened the inhibition of 15-LO by NFA compared to 9NOA. A similar structure–activity relationship was observed in the recombinant 5-LO enzyme activity assay.

The effects of NFA on COX-derived prostaglandin synthesis are complex and highly structure-dependent. NFA inhibit prostaglandin formation by lowering the heme group–binding stability (Bonilla et al., 2013; Wood et al., 2019). Nitrated AA could inhibit COX-1 but not COX-2 oxygenase activity, whereas it could inhibit the peroxidase activity in both isoforms (Maucher et al., 2017). However, Trostchansky *et al.* could not demonstrate inhibitory activity of 9NOA and nitro-linoleic acid on the peroxidase activity of both COX isoforms (Trostchansky et al., 2011). Additionally, in the present study, we were able to demonstrate that 9NOA and NFA 20.9.9, which has a similar chain length to the analyzed nitrated AA from Trostchansky et al. (2011), significantly inhibited COX-2 peroxidase activity. Hypothetically, the increased COX-2 activity induced by NFA 20.9.9, 16.9.9, and 14.9.9 may be based on the structural similarity to the COX substrate AA. However, NFA derivatives 12.9.9, 14.9.9, and 16.9.9 failed increasing COX-2 product formation in the cellular COX-2 assay (Supplementary Figure S5) leaving open the cause for the “negative” inhibition of rCOX-2. Next to the elongation of the chain length, the shift of the Michael acceptor moiety also led to improved COX-2 inhibition by NFA 18.10.10. Thus, COX-2 inhibition requires an accurately defined NFA structure with a certain chain length and Michael acceptor position. Notably, already small structural changes can have a large impact on the effect of NFA on COX-2. However, this complex structure–activity relationship remains unclear and requires further investigation. Moreover, NFA failed to inhibit COX-2 in cellular systems excluding these compounds as COX-2 inhibitors.

Recently, other research groups and ours described NFA-mediated anti-tumorigenic effects in animal models of breast cancer and colorectal cancer (Woodcock et al., 2018; Rom et al., 2019). Although the mode of action remains unclear, mitochondrial as well as proteasomal involvement is presumed. Again, short-chain NFA seem to have less anti-inflammatory efficacy but also less anti-tumorigenic efficacy (Table 5). 9NOA and NFA 20.9.9 were the most potent inducers of cytotoxicity in both HCT-116 and HT-29 cells and therefore have an optimum chain length. Relocation of the Michael acceptor moiety in NFA 18.7.7, 18.8.8, and 18.8.9 further increased the cytotoxic effect of 9NOA. Whereas NFA 18.7.7 had increased effects on a number of different anti-inflammatory signaling pathways, the biological effects of NFA 18.8.8 are mainly restricted to inhibiting NF-κB signaling and inducing cytotoxicity in colorectal cancer cells.

A recent study by Khoo *et al.* revealed the first insights into NFA structure–activity relationships regarding Nrf-2 and NF-κB signaling. Alterations in the fatty acid acyl chain length and Michael acceptor position correlated with Nrf-2 induction and NF-κB inhibition (Khoo et al., 2018). In detail, Khoo *et al.* proposed that increasing the omega acyl chain length enhances NFA Nrf-2 induction, whereas shortening the omega acyl chain end yielded stronger NF-κB inhibitors. We must, however, advise critical reflection, as the proposed effects were observed using luciferase-based measurements although the reduction of luciferase bioluminescence by fatty acids is a well-known side effect (Matsuki, 1999; Marques et al., 2015). Our studies confirm that lengthening the omega acyl chain end promotes Nrf-2 induced gene expression, where strong Nrf-2 induction is observed with up to omega-12 NFA. However, NFA with chains longer than 18 carbon atoms led to a slight decrease in pathway induction. On the other hand, contradictory results were observed for NF-κB signaling inhibition as the inhibition of this pathway not only depends on the NFA chain length and nitro group position but also on the double bond position, as seen for NFA 18.6.7, showing reduced NF-κB inhibition compared to NFA 18.7.7. Furthermore, only a distinct length of the NFA omega acyl chain end can inhibit p50 and p65 NF-κB signaling. We were unable to confirm the finding that short-chain NFA inhibit NF-κB signaling, as significant pathway inhibition was observed for only long-chain NFA, strengthening the hypothesis that luciferase-based pathway analysis is not suitable for use with NFA. Additionally, our structure–activity relationship study was extended to further NFA targets, including sEH, LOs, COX-2, and cytotoxicity studies on colorectal cancer cells to gain deeper insights into NFA multifunctional pharmacology.

From a (patho)physiological point of view we can conclude that shorter NFA metabolites generated from β-oxidation *in vivo* successively lose their biological activity as observed in our NF-κB, sEH, cytotoxicity and LOX-assays. Metabolism of NFA is rather complex and includes nest to β-oxidation also saturation leading to loss of electrophilicity and esterification into complex lipids (Buchan et al., 2018). Esterification of unsaturated NFA into complex lipids may however generate a sustained depot of potentially bioactive lipids, of which electrophilic NFA might be continuously released following acute or chronic inflammatory conditions (Buchan et al., 2018).

The classic drug development strategy is based on designing drugs affecting a single target. The target molecule can be an enzyme, transporter, or transcription factor (Giordano and Petrelli, 2008; Talevi, 2015). Research and development in the last few decades have focused on single-target drugs because they have assessable therapeutic effects, as well as manageable adverse effects. However, due to increasingly complex disease pathophysiology, not only a single pathway or organ system is affected. Multimorbidity causes a complex interaction of cardiovascular, pulmonary, and metabolic diseases, making necessary polypharmacology approaches potentially associated with unpredictable and partly injurious risks of drug–drug interactions (Garfinkel and Mangin, 2010; Divo et al., 2014; Busa et al., 2018). The increase in the incidence of comorbidities was the rationale for the design of multifunctional drugs, with increasing importance placed on covalent reactivity (Lera and Ganesan, 2016; Ramsay et al., 2018). As shown in the present study and by others, NFA are potent covalent protein modifiers affecting a number of pathophysiological relevant targets with favorable pharmacodynamic and pharmacokinetic properties in preclinical and clinical models (ClinicalTrials.gov Identifier: NCT02460146, NCT02248051, NCT03449524, NCT03422510). The multi-target activity of NFA implies therapeutic activity both in multimorbid patients and in patients with complex multifactorial proinflammatory diseases such as sepsis. Furthermore, their covalent reactivity with target proteins, observed in cell culture experiments and animal models of disease, implies sustained and efficient drug effects in patients (Piesche et al., 2020). Finally, as demonstrated in the present study, structural changes in NFA can considerably change target selectivity and the NFA pharmacodynamic profile. The design of NFA prodrugs with potentially increased membrane permeability e.g. due to esterification of the carboxyl group might be one of several future strategies to improve NFA pharmacokinetics and therapeutic efficacy. Future studies should further develop the active NFA derivatives identified in the present study to further increase potency, efficacy, and target selectivity. These NFA derivatives or mimetic may reach the status as drug candidate and then allow in-deep studies on selected optimized compounds using an animal model of inflammation.

Taken together, NFA are multi-target lipid mediators with strong therapeutic effects in preclinical models of disease such as local and systemic inflammation, tumorigenesis, and cardiovascular disorders. Their good tolerability in patients and their beneficial and structurally alterable pharmacodynamic profiles strongly suggest that NFA are promising drug candidates for further development. Future studies are needed to confirm the pharmacological effects of the different promising NFA derivatives proposed in this study both in preclinical models of disease and in clinical trials.

## Data Availability

The original contributions presented in the study are included in the article/Supplementary Material, further inquiries can be directed to the corresponding authors.

## Abbreviations

COX, cyclooxygenase; DHET, dihydroxyeicosatrienoic acid; EET, epoxyeicosatrienoic acids; GSH, glutathione; HETEs, hydroxyeicosatetraenoic acids; HO-1, heme oxygenase 1; Keap1, kelch-like ECH-associated protein 1; LO, lipoxygenase; LTB_4_, leukotriene B_4_; MMF, monomethyl fumarate; NFA, nitro-fatty acids; NF-κB, nuclear factor kappa B; Nrf-2, nuclear factor erythroid 2-related factor 2; NO, nitric oxide; nitro-AA, nitro-arachidonic acid; OA, oleic acid; PtGR-1, prostaglandin reductase 1; PKCα, protein kinase C alpha; PPAR, peroxisome proliferator-activated receptors; PGG_2_, prostaglandin G_2_; PGH_2_, prostaglandin H_2_; sEH, soluble epoxide hydrolase; TLR4, toll-like receptor 4; tBHQ, tert-butylhydroquinone; 9NOA, 9-nitro-oleic acid.
