# Supplementary material for: Structural Modifications Yield Novel Insights Into the Intriguing Pharmacodynamic Potential of Anti-inflammatory Nitro-Fatty Acids
Source: Front Pharmacol. 2021 Nov 18;12:715076. doi: 10.3389/fphar.2021.715076 (PMC8637440; doi:10.3389/fphar.2021.715076)
Supplement: Supplementary file 1 [file DataSheet1.pdf]

1 Supplementary Figures

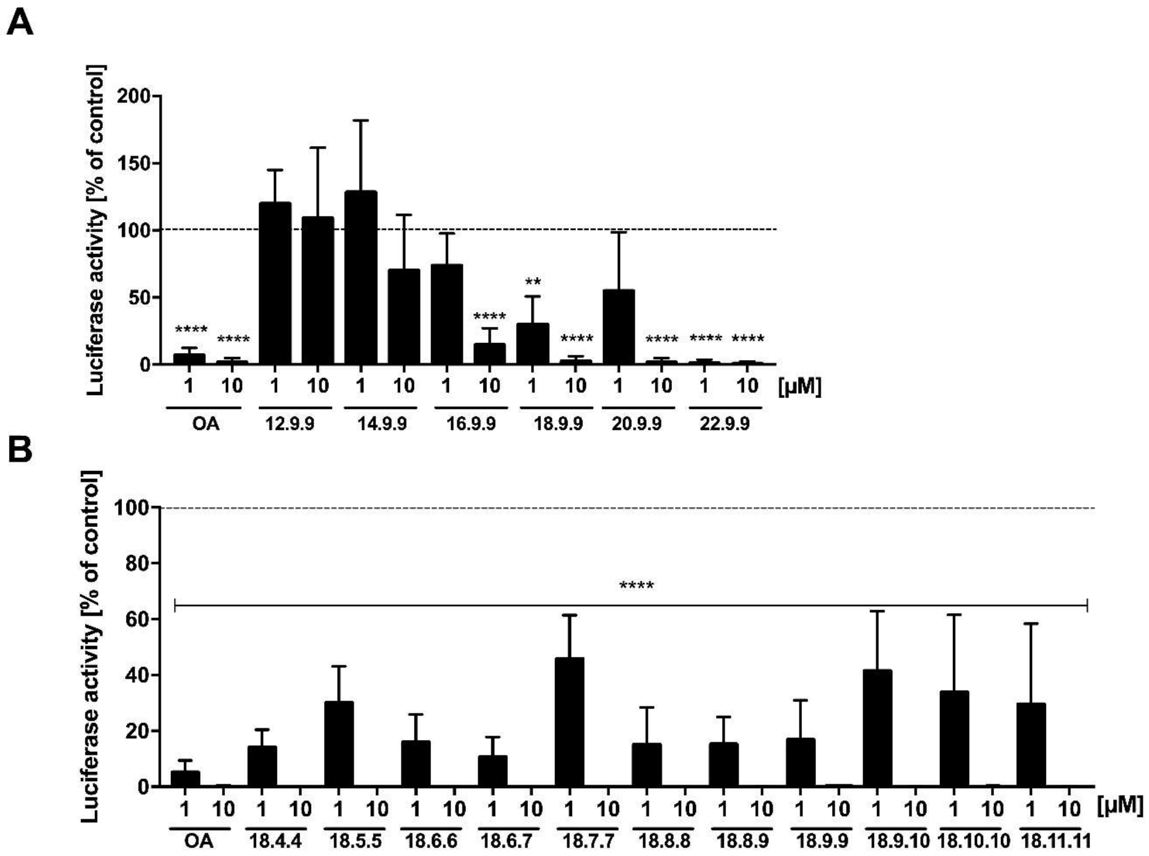

Suppl. Figure 1: Fatty acids interfere with firefly luciferase making the *luc*-based assays not suitable for analysis of signaling pathways modulated by NFA. 10^4 units of firefly luciferase were incubated with 1 µM and 10 µM OA or NFA with different carbon chain length (A) and Michael acceptor position (B) for 15 min at 37°C. Firefly activity is presented as percentage of vehicle control (DMSO) mean + SD, n=5. Compared to vehicle control statistical significance was calculated by one-way ANOVA with Bonferroni post-test. P-values of **P≦0.0021, **** P≦0.0001 are considered significant. *DMSO, dimethyl sulfoxide; 9NOA, 9-nitro oleic acid; NFA, nitro-fatty acids, OA, oleic acid.*

A

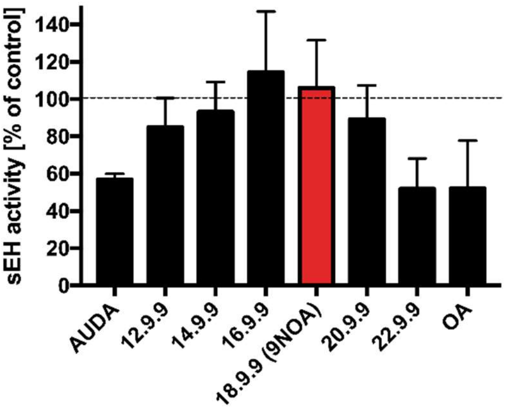

B

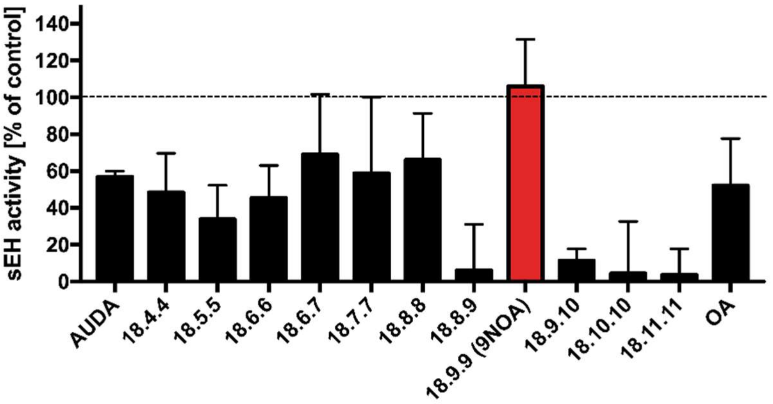

Suppl. Figure 2: Effects of NFA on sEH activity in COS-7 cells. sEH activity in COS-7 cells treated with NFA (10 µM) differ in (A) chain length and (B) Michael acceptor position and AUDA (10 µM) for 24h. Cells were lysed, and hydrolase activity was measured by Epoxy Fluor 7 lysis presented as mean + SD, n=2-3. *9NOA, 9-nitro oleic acid; NFA, nitro-fatty acids. OA, oleic acid, she, soluble epoxide hydrolase.*

A

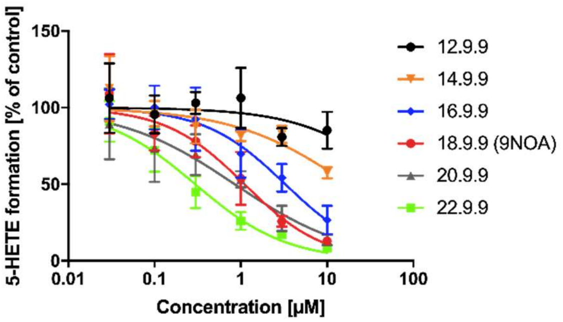

B

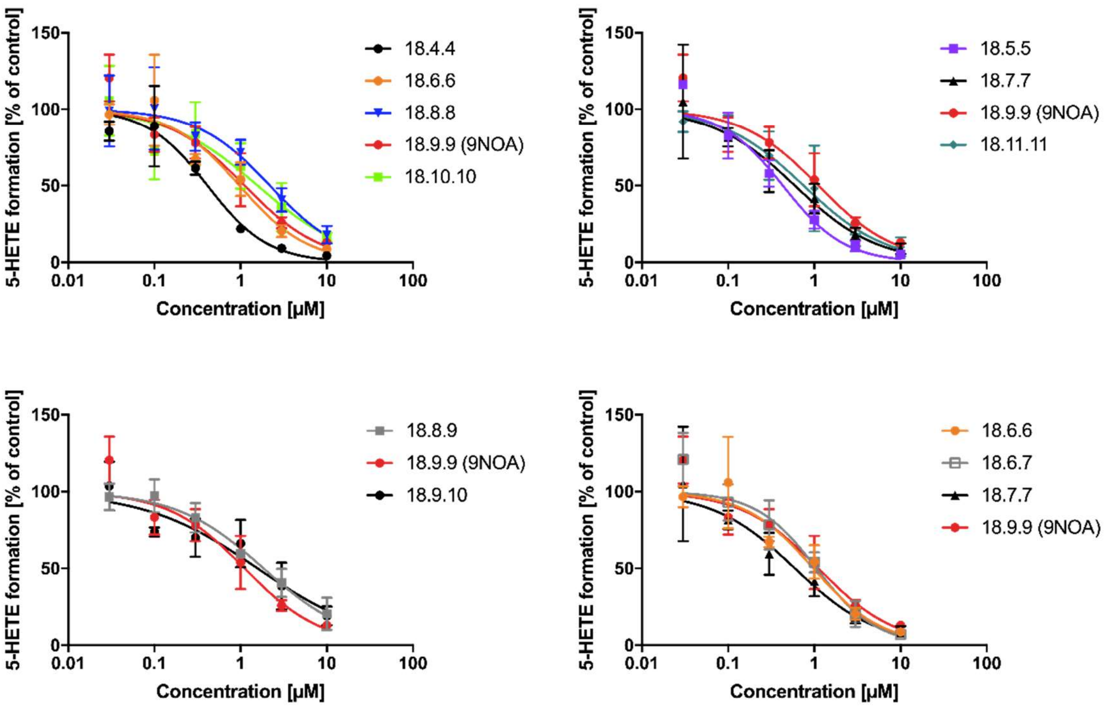

Suppl. Figure 3: Inhibition of 5-LO derived 5-HETE production by NFA with (A) different carbon chain lengths and (B) different Michael acceptor positions using the r5LO-wt assay. r5LO-wt (3 µg) in PBS/EDTA/ATP buffer was preincubated with the compounds or the vehicle control DMSO (0.1%) for 15 min at 4°C, preheated at 37°C for 30 sec, and subsequently stimulated with 1 mM $CaCl_2$ and 20 µM AA for another 10 min at 37°C. 5-HETE activity was analyzed by LC-MS. Data are presented as the percent of the control mean ± SD, n = 3–6. AA, *arachidonic acid;* ATP, *adenosine triphosphate;* DMSO, *dimethyl sulfoxide;* EDTA, *ethylenediaminetetraacetate;* HETE, *hydroxyeicosatetraenoic acid;* NFA, *nitro-fatty acids;* PBS, *phosphate-buffered saline.*

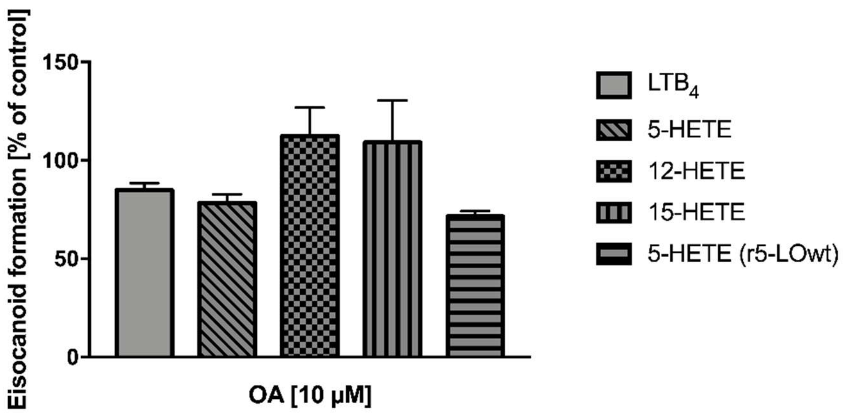

Suppl. Figure 4: Effect on leukotriene B_4 and eicosanoids by non-nitrated oleic acid in PMNL and r5LO-wt. PMNL were preincubated with 10 µM OA or vehicle control DMSO (0.1%) for 15 min at 37°C and subsequently stimulated with 2.5 µM Ionophore A23187 and 20 µM AA for another 10 min at 37°C. r5LO-wt in PBS/EDTA/ATP buffer were preincubated with compounds or vehicle control DMSO (0.1%) for 15 min at 4°C, preheated at 37°C for 30 sec and subsequently stimulated with 2 mM $CaCl_2$ and 20 µM AA for another 10 min at 37°C. LTB_4 and eicosanoid activity (5-/12-/15- HETE) was analyzed by LC-MS. Data are represented as percent of control mean + SD of n=3. AA*, arachidonic acid;* ATP*, adenosine triphosphate;* DMSO*, dimethyl sulfoxide;* EDTA*, ethylenediaminetetraacetate;* HETE*, hydroxyeicosatetraenoic acid;* LTB_4*, leukotriene B_4;* NFA*, nitro-fatty acids;* PBS, *phosphate-buffered saline;* r5LO-wt, *recombinant 5-lipoxygenase wild-type*

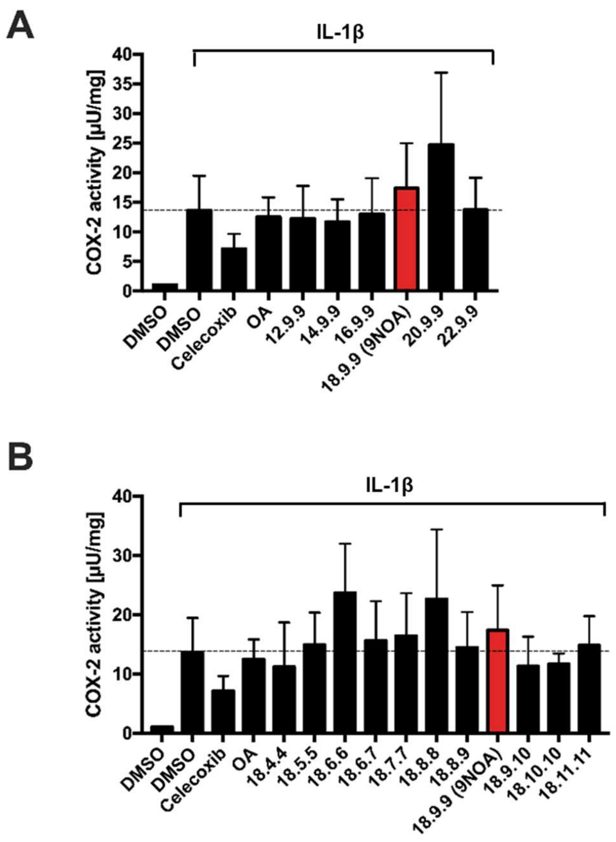

Suppl. Figure 5: Effects of NFA on cellular COX-2 activity. COX-2 Activity of A549 cells stimulated with IL-1β and subsequent treatment of NFA (10 µM) and Celecoxib (5 µM) for 24 h. Cells were lysed, and COX-2 activity was measured using Cyclooxygenase (COX) Activity Assay Kit (Fluorometric) from Abcam. (A) NFA derivatives with different chain length. (B) NFA derivatives with different Michael acceptor position. Samples were normalized to protein concentration. Data are presented as mean + SD of n=4. COX, *cyclooxygenase*; DMSO*, dimethyl sulfoxide* NFA, *nitro fatty acids*.

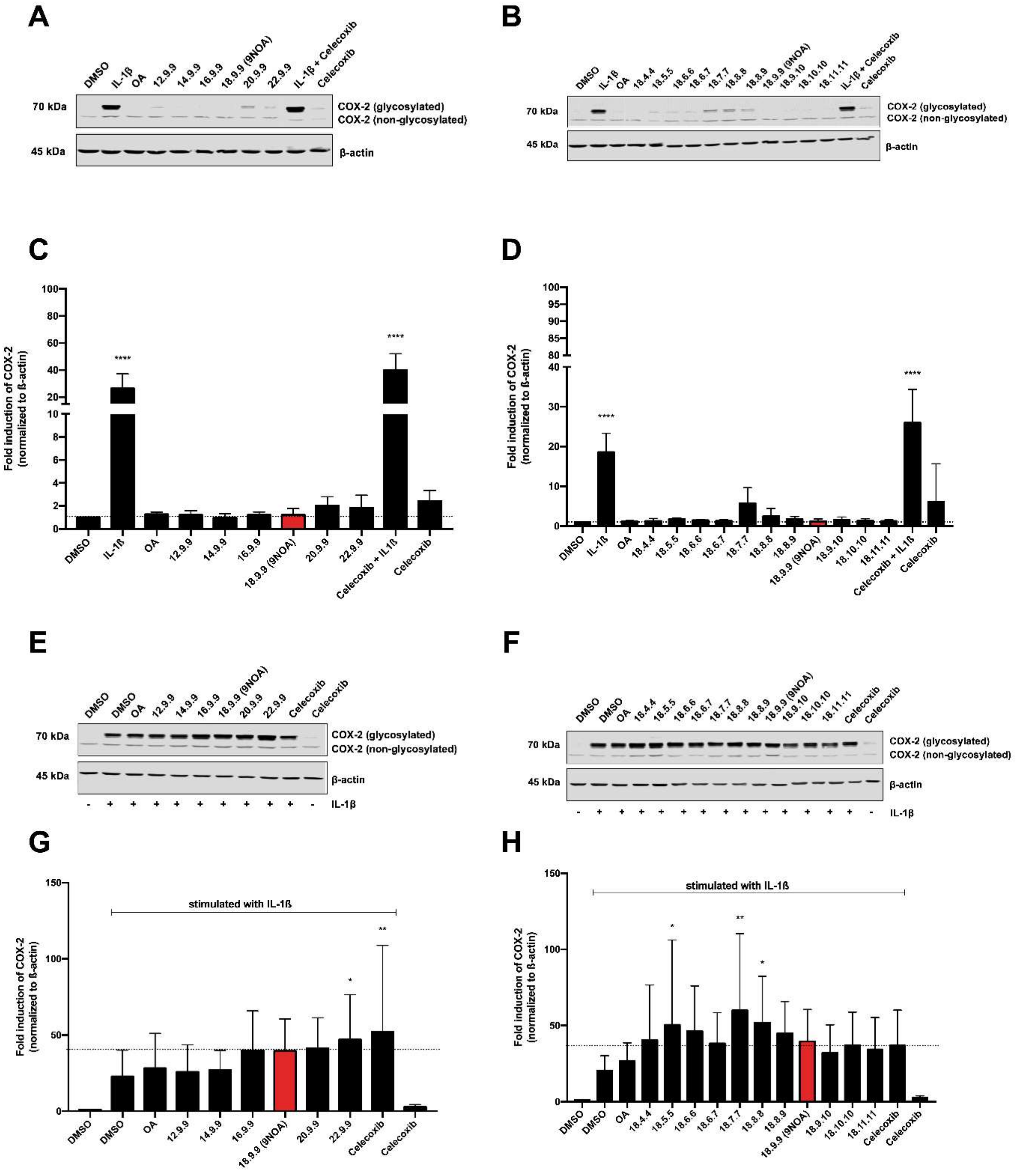

Suppl. Figure 6: Effects of NFA on cellular COX-2 expression. Representative Western Blot of A549 cells treated with (A) NFA with different carbon chain length and (B) different Michael acceptor position for 24 h without IL-1β stimulation (1 ng/ml) and the densitometrical evaluation of n=4-5 experiments (C, D). Representative Western Blot of A549 cells stimulated with IL-1β and subsequent treatment with (E) NFA with different carbon chain length and (F) different Michael acceptor position for 24 h and the densitometrical evaluation of n=4-5 experiments (G, H). Intracellular COX-2 levels were normalized to β-actin and presented as mean + SD. Statistical significance was calculated by one-way ANOVA with Bonferroni post-test. P-values of **** P≦0.0001, **P≦0.0021, and *P≦0.03 are considered significant. COX, *cyclooxygenase*; DMSO*, dimethyl sulfoxide;* NFA, *nitro fatty acids*

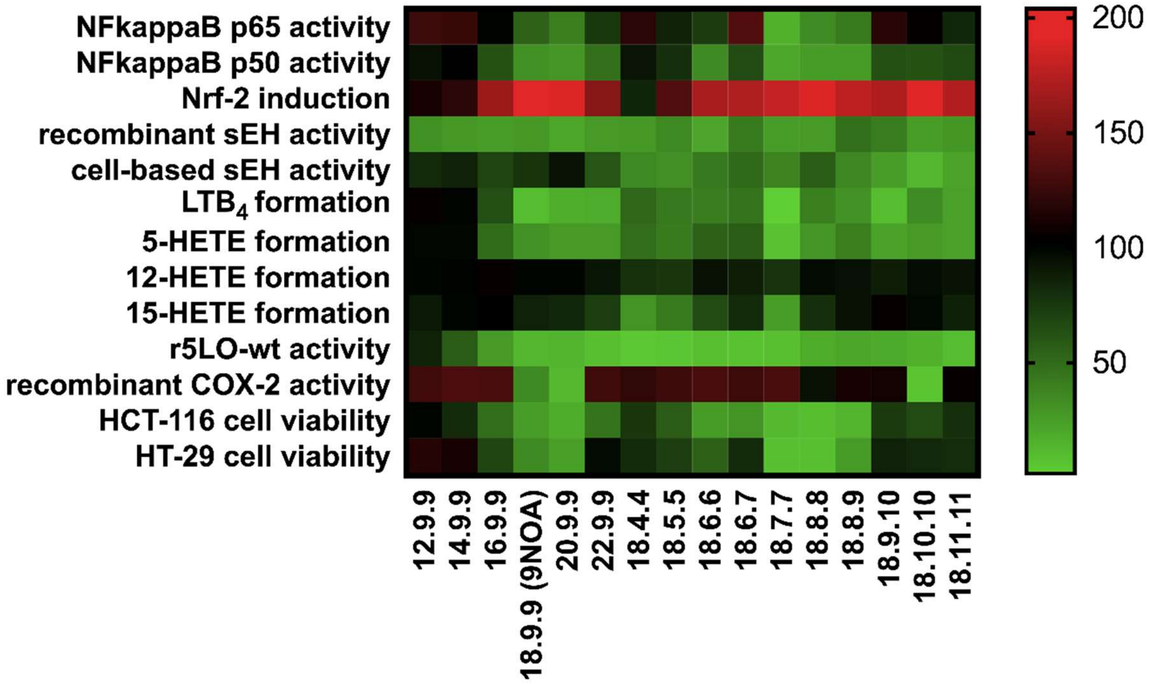

Suppl. Figure 7: Pathway heatmap depicts analyzed pathways upon NFA treatment. Effects on NF-κB inhibition; induction of Nrf-2; sEH, COX-2, and LO enzyme inhibition; as well as the cytotoxic effects on colorectal cancer cells were analyzed. Green indicates high inhibition, black indicates no significant effects, and red indicates high pathway induction. This view illustrates certain NFA chain lengths and Michael acceptor positions favorable in multiple pathways. COX-2, *cyclooxygenase-2,* HETE*, hydroxyeicosatetraenoic acid;* LTB_4*, leukotriene* B_4*;* NFA, *nitro-fatty acids*; NF-**κ**B, *nuclear factor B*; Nrf-2, *nuclear factor erythroid 2-related factor*; r5LO-wt: *recombinant 5-lipoxygenase wild-type*; sEH, *soluble epoxide hydrolase*;
